# Comparative Genomic Analysis of *Drechmeria coniospora* Reveals Core and Specific Genetic Requirements for Fungal Endoparasitism of Nematodes

**DOI:** 10.1371/journal.pgen.1006017

**Published:** 2016-05-06

**Authors:** Kevin Lebrigand, Le D. He, Nishant Thakur, Marie-Jeanne Arguel, Jolanta Polanowska, Bernard Henrissat, Eric Record, Ghislaine Magdelenat, Valérie Barbe, Sylvain Raffaele, Pascal Barbry, Jonathan J. Ewbank

**Affiliations:** 1 CNRS and University Nice Sophia Antipolis, Institute of Molecular and Cellular Pharmacology, Sophia Antipolis, France; 2 Centre d’Immunologie de Marseille-Luminy, Aix Marseille Université UM2, Inserm, U1104, CNRS UMR7280, Marseille, France; 3 CNRS UMR 7257, Aix-Marseille University, Marseille, France; 4 INRA, USC 1408 AFMB, Marseille, France; 5 Department of Biological Sciences, King Abdulaziz University, Jeddah, Saudi Arabia; 6 INRA, UMR1163 Biodiversité et Biotechnologie Fongiques, Aix-Marseille Université, Polytech Marseille, CP 925, Marseille, France; 7 Aix-Marseille Université, UMR1163 Biodiversité et Biotechnologie Fongiques, Faculté des Sciences de Luminy-Polytech, CP 925, Marseille, France; 8 Commissariat à l'Energie Atomique, Institut de Génomique, Génoscope, Laboratoire de Biologie Moleculaire pour l'Etude des Génomes (LBioMEG), Evry, France; 9 INRA, Laboratoire des Interactions Plantes-Microorganismes (LIPM), UMR441, Castanet Tolosan, France; 10 CNRS, Laboratoire des Interactions Plantes-Microorganismes (LIPM), UMR2594, Castanet Tolosan, France; MicroTrek Incorporated, UNITED STATES

## Abstract

*Drechmeria coniospora* is an obligate fungal pathogen that infects nematodes via the adhesion of specialized spores to the host cuticle. *D*. *coniospora* is frequently found associated with *Caenorhabditis elegans* in environmental samples. It is used in the study of the nematode’s response to fungal infection. Full understanding of this bi-partite interaction requires knowledge of the pathogen’s genome, analysis of its gene expression program and a capacity for genetic engineering. The acquisition of all three is reported here. A phylogenetic analysis placed *D*. *coniospora* close to the truffle parasite *Tolypocladium ophioglossoides*, and *Hirsutella minnesotensis*, another nematophagous fungus. Ascomycete nematopathogenicity is polyphyletic; *D*. *coniospora* represents a branch that has not been molecularly characterized. A detailed *in silico* functional analysis, comparing *D*. *coniospora* to 11 fungal species, revealed genes and gene families potentially involved in virulence and showed it to be a highly specialized pathogen. A targeted comparison with nematophagous fungi highlighted *D*. *coniospora*-specific genes and a core set of genes associated with nematode parasitism. A comparative gene expression analysis of samples from fungal spores and mycelia, and infected *C*. *elegans*, gave a molecular view of the different stages of the *D*. *coniospora* lifecycle. Transformation of *D*. *coniospora* allowed targeted gene knock-out and the production of fungus that expresses fluorescent reporter genes. It also permitted the initial characterisation of a potential fungal counter-defensive strategy, involving interference with a host antimicrobial mechanism. This high-quality annotated genome for *D*. *coniospora* gives insights into the evolution and virulence of nematode-destroying fungi. Coupled with genetic transformation, it opens the way for molecular dissection of *D*. *coniospora* physiology, and will allow both sides of the interaction between *D*. *coniospora* and *C*. *elegans*, as well as the evolutionary arms race that exists between pathogen and host, to be studied.

## Author Summary

Some soil-living fungi can kill nematodes and are used as biocontrol agents against plant parasitic nematodes. Certain species trap their prey using adhesive knobs or nets. For others, like *Drechmeria coniospora*, infection starts with the adhesion of specialized non-motile spores to the nematode cuticle. We have sequenced and annotated the *D*. *coniospora* genome. Comparative and functional genomic analyses provide insights into how its nematode-destroying lifestyle has evolved. We identified genes that were found only in *D*. *coniospora*, others found only in nematophagous species; many were highly expressed and differentially regulated during the different stages of fungal growth or during nematode infection. We have also developed methods for the genetic modification of *D*. *coniospora* that can be used to probe the function of its genes, allowing the dissection of this mode of nematode killing. We used them to probe a specific interaction between *D*. *coniospora* and *C*. *elegans*, involving the potential interference by the pathogen of a host antimicrobial mechanism.

## Introduction

Species of nematophagous fungi have evolved a variety of strategies to invade and kill their nematode hosts in order to use them as a source of nutrients. Some species, exemplified by *Arthrobotrys oligospora*, form specialized and elaborate hyphal structures that trap nematodes, while others, like *Monacrosporium haptotylum*, use adhesive branches [1]. In addition to the fundamental interest in understanding these remarkable adaptations, these fungi are of economic importance because of their long-recognized potential as biocontrol agents of plant parasitic nematodes [2]. Insights into the molecular mechanisms that underlie the virulence of nematophagous species, and into their evolution, have been obtained from a series of genomic analyses (e.g. [3–9]).

*Drechmeria coniospora* produces non-motile spores (conidia) that stick to the nematode cuticle, via a specialized adhesive bud [10, 11]. Shortly after, the spores germinate, producing an appresorium that allows the fungus to pierce the nematode cuticle and send hyphae into its epidermis [10]. Until now, there has been essentially no molecular characterisation of *D*. *coniospora*. Thus, at the start of this project, nothing was known about its genetic makeup, apart from 1.05 kb of rRNA sequence in Genbank (GI:16763389; AF106012) that had been used to assign *D*. *coniospora* to the hypocrealean family, Clavicipitaceae, which includes many fungal pathogens of arthropods, such as *Beauveria bassiana* [12]. The same single sequence was used in a subsequent analysis that removed *Drechmeria* from the Clavicipitaceae and recognized it as one of six genera within the Ophiocordycipitaceae [13].

*D*. *coniospora* was adopted as a model fungal pathogen of *C*. *elegans* 30 years ago. Since the first studies in this domain [14, 15], *C*. *elegans* has emerged as a powerful model system for the investigation of host-pathogen interactions [16–22], and *D*. *coniospora* shown to be a natural pathogen of *C*. *elegans* [23]. We have put considerable effort into understanding the host defences that are triggered by *D coniospora* infection (e.g. [24–28]). Great strides in dissecting host defences in other organisms have been gained by investigating how pathogens evade or subvert these mechanisms (e.g. [29–32]). Understanding what is happening on the pathogen side during infection in the *D*. *coniospora-C*. *elegans* model could therefore be key to unravelling completely the host defence network, especially as the two protagonists are likely to have co-evolved [23, 33].

Completing a high-quality draft genome of *D*. *coniospora* is a very useful first step for understanding its virulence mechanisms. Combined with RNAseq transcriptomic and *in silico* analyses, it allowed us to predict a first complete gene set for *D*. *coniospora*. A comparison with other fungi, including nematode-destroying species, has revealed genes potentially involved in virulence and given insights into the evolution of the infectious capability of *D*. *coniospora*. To be able to exploit this knowledge, we established a method for fungal transformation, and, in a proof-of-principle, used it to generate recombinant knock-out and knock-in strains. These different approaches allowed us to initiate the investigation of a potentially novel fungal counter-defensive strategy.

## Results

### Sequencing output processing, *de novo* genome assembly and scaffolding

*D*. *coniospora* genomic DNA was sequenced on an Illumina MIseq sequencer as 2 x 150 bp paired-end reads. After filtering, we obtained 11.3 million reads, for a 100X coverage of a genome originally estimated to be 30 Mb. To determine which frequently used *de novo* genome assembly program performed best with this set of data, we tested four, Velvet [34], SPAdes [35], SOAPdenovo2 [36] and ABySS [37]. Each assembly was scaffolded with SSPACE [38], using two libraries of mate-paired 2 x 60 bp SOLiD reads. We applied a stringent filter, keeping only very high-quality reads, to limit errors during scaffolding. The libraries finally contained 23.2 and 23.6 million mate-paired reads, with insert sizes of 1.5 kb and 3 kb, respectively. Two contigs were scaffolded, using SSPACE, only when supported by at least 5 shared mate-paired reads. The overall characteristics of the *de novo* genome assemblies are shown in Table 1, before and after the SSPACE scaffolding step. ABySS (with kmer 96) and SPAdes performed well, giving low numbers of both contigs and unknown nucleotides, with ABySS maximizing the N50 value (2.09 Mb; length for which the collection of contigs of that length or longer contains half the total length of all contigs).

**Table 1:** Descriptive statistics of the different assemblies before and after SSPACE scaffolding.

	Before SSPACE					After SSPACE				
	Vel	So	Sp	A64	A96	Vel	So	Sp	A64	A96
**Number contigs**	455	12451	1075	7880	1356	190	11700	470	7157	1023
**Total length (Mb)**	31.6	33.1	31.9	32.3	32.0	31.7	34.0	31.9	32.8	32.1
**Max contig length (Mb)**	1.27	1.94	0.51	1.36	1.20	3.00	4.54	4.50	2.83	4.80
**N50 (Mb)**	0.32	0.58	0.12	0.35	0.24	1.40	1.76	1.21	1.70	2.09
**% of Ns**	0.77	1.85	0.00	0.24	0.12	1.13	4.58	0.16	1.85	0.33

Total number of contigs; total size of the assembly; length of the longest contig; N50; percentage of unknown bases. Values are given for each assembler (Vel: Velvet with kmer = 63, So: SOAPdenovo with kmer = 63, Sp: Spades with kmer = 127; A64 and A96: ABySS with kmer = 64 and 96, respectively) before and after scaffolding with SSPACE.

### Optical mapping data integration

We then used optical mapping to test further the quality of the different assemblies generated before and after SSPACE scaffolding. Individual chromosomes were stretched on a glass slide and cut *in situ* with a restriction enzyme. The resulting fragments were visualized using a fluorescent microscope and their lengths measured. These lengths were compared to the predicted lengths of fragments from the longer scaffolds (i.e. with a length of at least 20 kb). The same approach was applied for each assembly. The nine distinct maps that were identified by optical mapping are indicative of a genome organization into 9 distinct chromosomes, with sizes ranging from 0.58 to 11.3 Mb (S1 Table).

**Fig 1:**
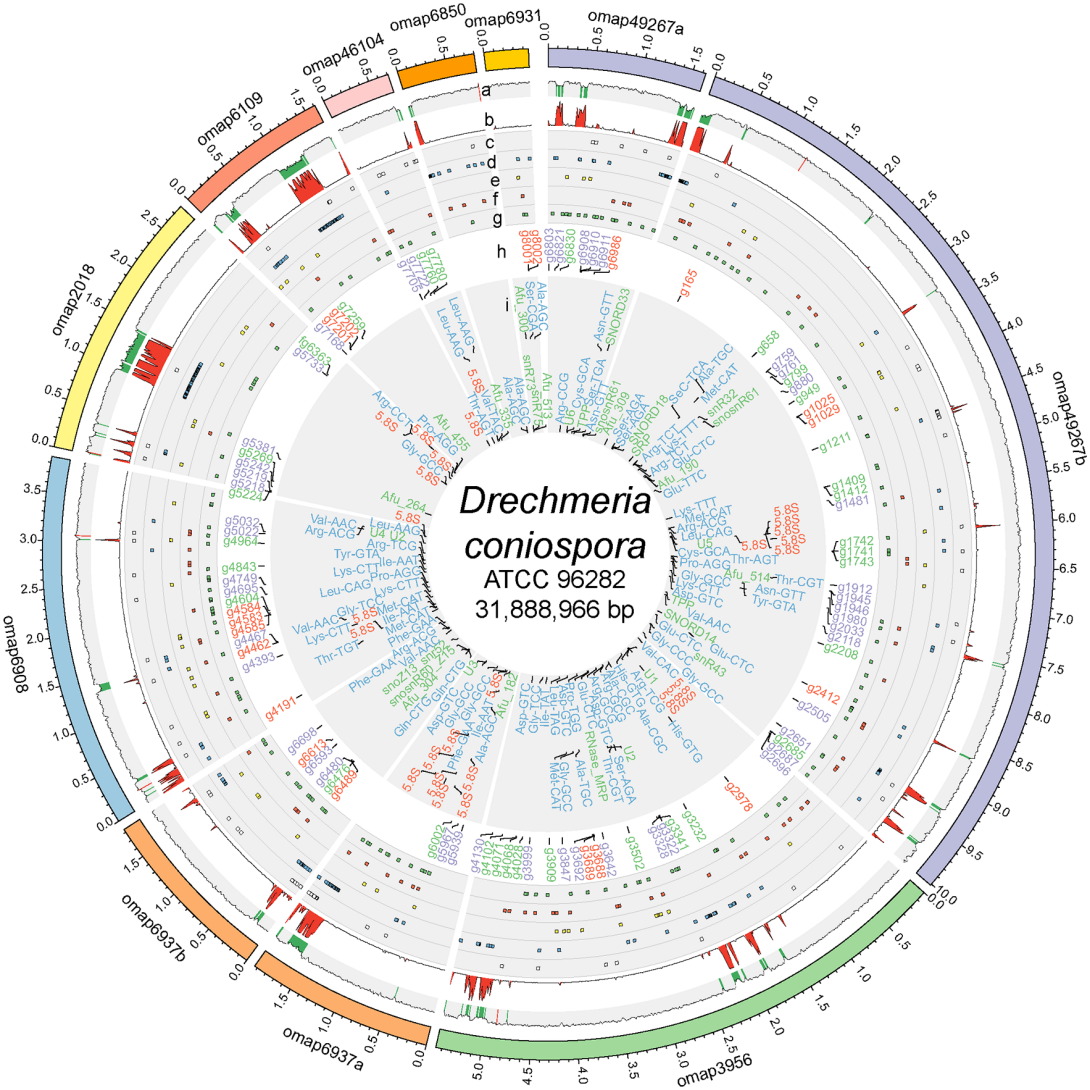
The *Drechmeria coniospora* genome. The optical maps (omap), potentially corresponding to distinct chromosomes, are depicted using Circos [39] with coloured sectors on the outer layer. As explained in the text, the optical maps 6937 and 49267 are split into two pieces. Scaffold43, corresponding to 23.8kb of mitochondrial DNA, and a further 63 other unanchored scaffolds, including 41 containing at least one predicted gene, and totalling 755,493 bp of genomic sequence, are not printed on the Circos plot. Each layer depicts, from the outside to the inside: **(a)** Percentage of G+C (red > 0.65, green < 0.45); **(b)** Percentage of repeat elements (red > 10%); **(c and d)** CLASS II and CLASS I transposable elements (white and blue blocks, respectively); **(e)** Members of three superfamilies encoding glutathione-S-transferases (GSTs), cytochrome P450 monooxygenases (P450s) and carboxyl/cholinesterases (CCEs) important for xenobiotic detoxification and oxidative stress resistance in entomopathogenic species [40] are depicted in yellow; **(f)** TM7 transmembrane proteins (red); **(g)** ABC proteins (green); **(h)** groups of genes discussed in the text that encode: putative nonribosomal peptide synthetases (red), diverse proteases (purple) and enterotoxin-like proteins in green (their names without the.t1 suffix are shown); **(i)** non-coding RNA genes: tRNAs (blue), rRNAs (red), others (green).

The assembly obtained with ABySS (kmer = 96) was selected since it maximized the remapping of the scaffolds on the optical map whilst at the same time minimizing the number of mis-assemblies observed in the optical map analysis (4 versus 20 for Spades and 9 for ABySS at a kmer value of 64; S1 Table). This low occurrence of scaffolding errors following SSPACE indicates that the first scaffolding step based on SOLiD reads would probably already have been of sufficient quality for an accurate assembly. During optical map analysis integration, we decided not to join scaffolds located on the same optical map when separated by a gap larger than 150 kb. Rather, for omap6937 and omap49267, the resulting map was divided into sub-chromosomal sequences. For instance, omap49267, the longest map, has a length of 11.3 Mb. It is represented in the final assembly by 2 sub-chromosomal sequences: omap49267a, with a length of 1.6 Mb and omap49267b, with a length of 10 Mb. The discrepancy in size between the sum of the lengths of the 2 sub-chromosomal sequences and that of omap49267 (11.6 Mb instead of 11.3 Mb) is explained by the fact that we did not break the scaffolds’ distal extremities during scaffold concatenation. It is noteworthy that the sequences both at the 3’ end of omap49267a and the 5’ end of omap49267b (i.e. within the corresponding chromosome) are of low complexity (Fig 1), which can explain the difficulty in mapping properly these extremities on the optical map. We then put aside all contigs smaller than 0.5 kb (a collection totalling 128 kb). The final genome assembly therefore includes 75 sequences for a total size of 31.9 Mb. It contains less than 0.2% of unknown nucleotides, and its N50 value is equal to 3.86 Mb. The combination of 2 types of short-read sequencing (SOLiD and MISeq) with optical mapping therefore provided the basis for a high-quality assembly of the *D*. *coniospora* genome.

### Transposable elements, repeat sequences, tRNAs, rRNAs and mtDNA

Amplification of transposable elements (TE) can contribute to chromosomal rearrangements, altering genomic structure and gene expression. It is believed to be an important route to speciation in some fungi. We therefore characterized the set of TEs predicted in the *D*. *coniospora* genome using TransposonPSI (http://transposonpsi.sourceforge.net/). A total of 600 elements were detected, falling into 12 classes (Table 2), and covering 1.6% of the genome (516 kb).

**Table 2:** Transposable elements in the *D*. *coniospora* genome.

Class 1 DNA transposons, LTR retroelements		Class 2 DNA transposons			
Name	Number	Name	Number	Name	Number
TY1_Copia	193	hAT	38	MuDR_A_B	15
gypsy	193	cacta	41	piggybac	2
DDE_1	46	mariner	23	helitronORF	2
LINE	26	mariner_ant1	20	ISC1316	1

We compared these results with the number of TEs found in the genomes of 11 other fungi, chosen on the basis of their phylogenetic position and/or lifestyle, using the name assigned by the NCBI Taxonomy Database: (1) *Arthrobotrys oligospora*, *Hirsutella minnesotensis*, *Monacrosporium haptotylum* and *Pochonia chlamydospora*, 4 nematophagous fungi that feed on different species and stages of nematode worms; (2) the entomopathogenic fungi *Metarhizium acridum*, *Metarhizium anisopliae* and *Ophiocordyceps sinensis*; (3) the plant pathogens *Fusarium graminearum* and *Fusarium oxysporum*; (4) the mycoparasite *Tolypocladium ophioglossoides*; (5) *Trichoderma reesei*, a model mesophilic and filamentous fungus. Although being numerous in *D*. *coniospora*, the number of TEs was in no way exceptional. For example, there has been a remarkable proliferation of retrotransposons in *Ophiocordyceps sinensis* [41]; its genome is predicted to contain 12862 TEs (S2 Table).

As part of its overall characterisation, we mined the *D*. *coniospora* genome for repetitive elements using RepeatScout [42]. The resulting specific repeat library was used for masking the genome with RepeatMasker (www.repeatmasker.org) and finally covered 2.73 Mb (8.6%) of the full genome. Genes for ribosomal 28S and 18S RNAs were identified on scaffold58 (6.3 kb), near a 5.8S rRNA subunit gene. In addition, 26 copies of 5S RNAs were detected scattered throughout the entire genome. A further 33 non-coding, non-tRNAs/rRNAs were detected. We also searched for predicted tRNAs and found a total of 123 nuclear tRNA loci (Fig 1). Eighteen mitochondrial-specific tRNAs were located on Scaffold43 (23.8 kb, G+C 25.7%) after a tRNAscan search [43] with the option “organelle”. The 141 predicted tRNAs correspond to 49 out of 64 codons. This degree of coverage compares very favourably with other sequenced fungal genomes (S3 Table). A more comprehensive investigation of Scaffold43 with TBLASTX [44] against a database of mitochondrial genes led to the identification of homologues for multiple mitochondrial proteins and established it as the mitochondrial DNA (S1 Text).

### Gene prediction and genome annotation

From the genomic DNA, Augustus [45] was used to predict the coding DNA sequence (CDS) for a total of 8733 genes, with a mean length of 1425 bp and a GC content of 61.3% (higher than for the overall genome sequence, as is generally the case). The predicted set represents a total of 12.4 Mb of coding nucleotides. To assess the quality and completeness of the prediction, we performed a BUSCO analysis [46], which is based on expectations of gene content from near-universal single-copy orthologs (USCOs). Using a set of more than 1400 fungal USCOs, again the annotation of the *D*. *coniospora* genome appeared to be of high quality, at least as good as that of the other fungi used in this study (Table 3).

**Table 3:** Universal single-copy ortholog prediction in *D*. *coniospora* and 11 other species.

	Complete single-copy	Complete duplicated	Fragmented	Missing	Source^a^
***Drechmeria coniospora***	98% (1412)	10% (153)	1.5% (22)	0.2% (4)	This study; PRJNA269584
***Trichoderma reesei***	97% (1407)	11% (167)	1.6% (24)	0.4% (7)	Trire2/Trire2.home.html
***Fusarium graminearum***	97% (1406)	11% (167)	2% (29)	0.2% (3)	Fusgr1/Fusgr1.home.html
***Arthrobotrys oligospora***	97% (1404)	11% (159)	2.1% (31)	0.2% (3)	Artol1/Artol.home.html
***Monacrosporium haptotylum***	96% (1390)	10% (156)	2.7% (39)	0.6% (9)	Monha1/Monha1.home.html
***Hirsutella minnesotensis***	96% (1388)	12% (183)	2.3% (34)	1.1% (16)	PRJNA67943
***Fusarium oxysporum***	96% (1386)	35% (511)	3.2% (47)	0.3% (5)	Fusox1/Fusox1.home.html
***Metarhizium anisopliae***	95% (1376)	11% (165)	3.5% (51)	0.7% (11)	Metan1/Metan1.home.html
***Tolypocladiumophioglossoides***	95% (1374)	10% (157)	1.6% (24)	2.7% (40)	PRJNA91059
***Metarhizium acridum***	95% (1370)	10% (152)	4% (58)	0.6% (10)	Metac1/Metac1.home.html
***Pochonia chlamydosporia***	88% (1270)	11% (168)	9% (130)	2.6% (38)	www.fungalinteractions.org/index.php/en/genome
***Ophiocordyceps sinensis***	71% (1026)	7.3% (105)	10% (152)	18% (260)	PRJNA59569

The table shows the percentage of the different categories of USCOs, with the corresponding number of proteins in brackets, as calculated by BUSCO. Orthologs are classified as ‘complete’ when their lengths are within two standard deviations of the BUSCO group mean length, otherwise they are classified as ‘fragmented’ (length not within the threshold) or ‘missing’. ‘Complete’ orthologs found with more than one copy are classified as ‘duplicated’.

^a^NCBI bioproject number, full URL, or end of URL at genome.jgi.doe.gov

With these results in mind, it is therefore interesting to compare this first comprehensive prediction for the *D*. *coniospora* genome with those of the other 11 fungi (Table 4).

**Table 4:** General characteristics of the *D*. *coniospora* genome compared to other species.

	*Drechmeria coniospora*	*Metarhizium acridum*	*Metarhizium anisopliae*	*Pochonia chlamydosporia*	*Arthrobotrys oligospora*	*Monacrosporium haptotylum*	*Fusarium graminearum*	*Fusarium oxysporum*	*Trichoderma reesei*	*Tolypocladium ophioglossoides*	*Hirsutella minnesotensis*	*Ophiocordyceps sinensis*
Sequencing depth (fold coverage)	100	107	100	136	37	28	85	-	48	76	128	241
Genome size (Mb)	31.9	39.4	39.2	42.4	40.1	39.5	36.5	61.4	33.5	31.2	51.1	78.5
Chromosomes^a^	9 + MT	-	-	-	-	-	4 + MT	15 + MT	7 + MT	-	-	-
GC content (%)	55.2	49.9	51.5	49.9	44.5	45.3	48.3	48.4	52.8	57.3	52.1	43.5
# of scaffolds	75	241	176	956	215	1279	31	114	87	172	736	10603
Scaffold N50 (Mb)	3.86	0.33	1.96	0.23	2.04	0.19	5.35	1.98	1.22	0.67	0.38	0.01
% of unknown (N's)	0.2	3.49	0.27	2.91	0.27	0.09	0.61	2.32	0.14	3.15	2.44	5.77
No. predicted protein-coding genes	8733	9849	10583	11079	11479	10959	13322	17708	9849	9317	12700	6972
Average exons per gene	2.83	2.7	2.68	-	3.17	3.31	2.82	2.7	2.88	-	2.5	-
Average exon length (bp)	504	549	568	-	473	470	508	498	512	-	-	-
Median protein length (aa)	393	406	416	393	407	416	366	366	408	410	403	362
Mitochondrial DNA (kb) ^a^	23.8	145	24.7	25.6	170.4	140.2	107.7	84.8	42.1	-	40.6	42.2
Repetitive sequence in Mb (%)	2.73 (8.6)	1.16 (2.9)	0.59 (1.5)	0.06 (0.1)	0.54 (1.4)	0.73 (1.9)	0.35 (1.0)	11.85 (19.3)	0.17 (0.5)	0.37 (1.2)	13.22 (25.9)	51.64 (65.8)
Transposable elements in Mb (%)	0.52 (1.6)	0.15 (0.4)	0.33 (0.8)	0.14 (0.3)	0.17 (0.4)	0.32 (0.8)	0.04 (0.1)	3.176 (5.2)	0.11 (0.3)	0.07 (0.2)	3.64 (7.1)	8.51 (10.8)
NCBI accession	PRJNA269584	ADNI00000000.1	PRJNA156697	AOSW00000000	ADOT00000000.1	AQGS00000000.1	AACM00000000.2	AAXH01000000	PRJNA118357	PRJNA91059	PRJNA67943	PRJNA59569

^a^ See S1 Text

*D*. *coniospora* was recently reassigned to the family Ophiocordycipitaceae [13]. Overall, compared to the 3 other family members included in our analysis, the *D*. *coniospora* genome much more closely resembles that of *Tolypocladium ophioglossoides* than either *Hirsutella minnesotensis* or *Ophiocordyceps sinensis*, in terms of size, GC content and the number of predicted genes. Both species have comparatively small genomes but a relatively high complement of predicted protein-coding genes.

### Phylogeny

**Fig 2:**
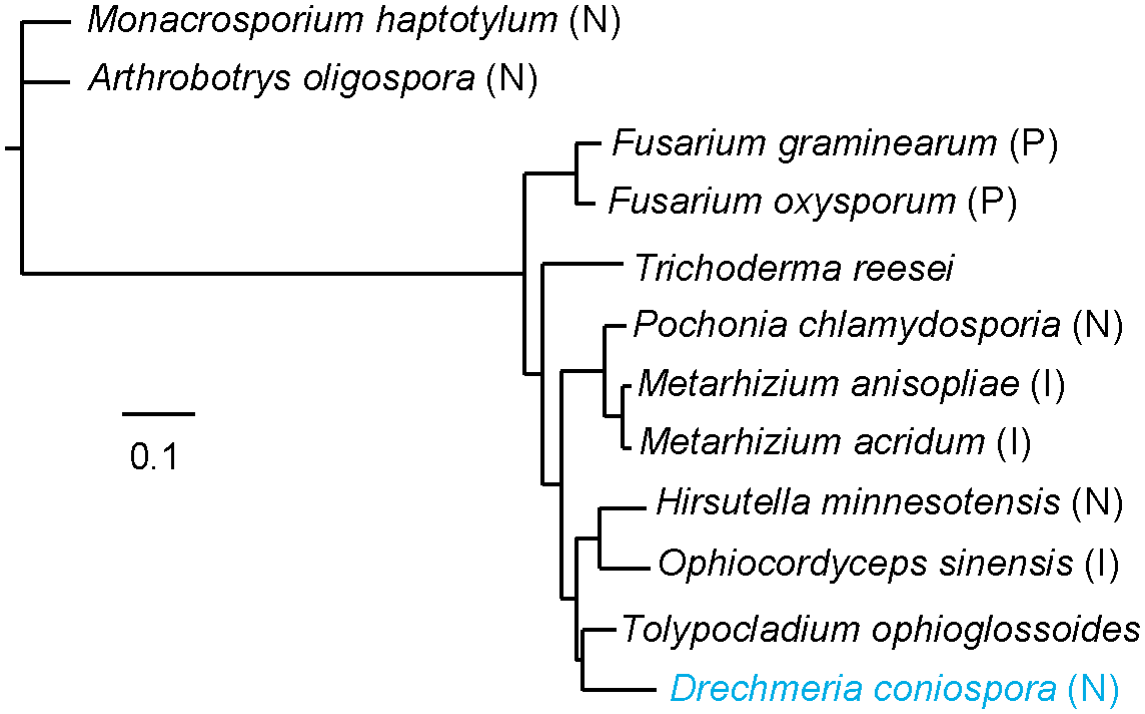
Phylogenetic tree for 12 Pezizomycotina fungi. Phylogenetic tree for 12 species based on alignments for a concatenation of 97 conserved protein sequences. Branch-lengths are drawn in proportion to the estimated number of substitutions per site. Species known to infect insects (I), nematodes (N) and plants (P) are indicated. All branches are fully supported (100/100 bootstraps).

A previous study placed *D*. *coniospora* in Ophiocordycipitaceae on the basis of a single DNA sequence [13]. In order to carry out a more thorough phylogenetic analysis, using BUSCO, we identified a set of 97 high-confidence orthologous proteins present in all 12 fungal species. Concatenated sequences (S4 Table) were aligned using MAFFT [47] and phylogenetic distances calculated using PhyML [48]. The overall phylogeny was in line with recent phylogenetic studies of *H*. *minnesotensis* and *P*. *chlamydosporia* [9, 49], and confirmed *D*. *coniospora*’s place in the Ophiocordycipitaceae family. Consistent with the general features of their respective genomes, the analysis placed *D*. *coniospora* closest to *T*. *ophioglossoides* (Fig 2). The results support the conjecture that in Hypocreales invertebrate-pathogenic fungi form a monophyletic group, distinct from cellulolytic, plant pathogenic filamentous fungi [49], while also providing a further illustration of the multiple independent origins of nematode pathogenic fungi and the distinct evolutionary trajectories of the trapping fungi such as *Arthrobotrys oligospora*, as opposed to the conidial species including *D*. *coniospora*.

### Functional annotation of the predicted proteome

In order to obtain a first overview of the set of proteins predicted from the *D*. *coniospora* genome, we functionally annotated the protein sequences with InterproScan [50]. This assigned at least one annotation to more than three quarters of them (6734/8733; 77.1%). There was a bias in the distribution of annotations; longer proteins were more likely to have an annotation. Thus while the vast majority (90.7%) of proteins as long or longer than the median (393 amino acids) had an annotation, only 63.4% of proteins shorter than the median had one. For smaller proteins, the effect was even more marked. Indeed, only half (50.6%) of proteins shorter than 250 amino acids long had an annotation (S5 Table). In line with previous observations [51], this bias was mirrored in the pattern of conservation. While overall, 60% (5248/8733) of the predicted proteins have a homologue in the curated UniprotKB/Swissprot database, 74.8% of the proteins as long or longer than the median were assigned a homologue, while only 34.6% of the proteins less than 250 amino acids were. It should be noted that these are conservative estimates of homology since although of high quality, the UniprotKB/Swissprot database is not exhaustive. Indeed, for example, on the basis of BLASTP analyses using the current NCBI non-redundant database, many (19/31) of the predicted proteins longer than 1000 amino acids but without any annotation have homologues in other fungi. The closest homologue was most often found in *Tolypocladium ophioglossoides* (S5 Table), consistent with the phylogenetic analysis.

**Fig 3:**
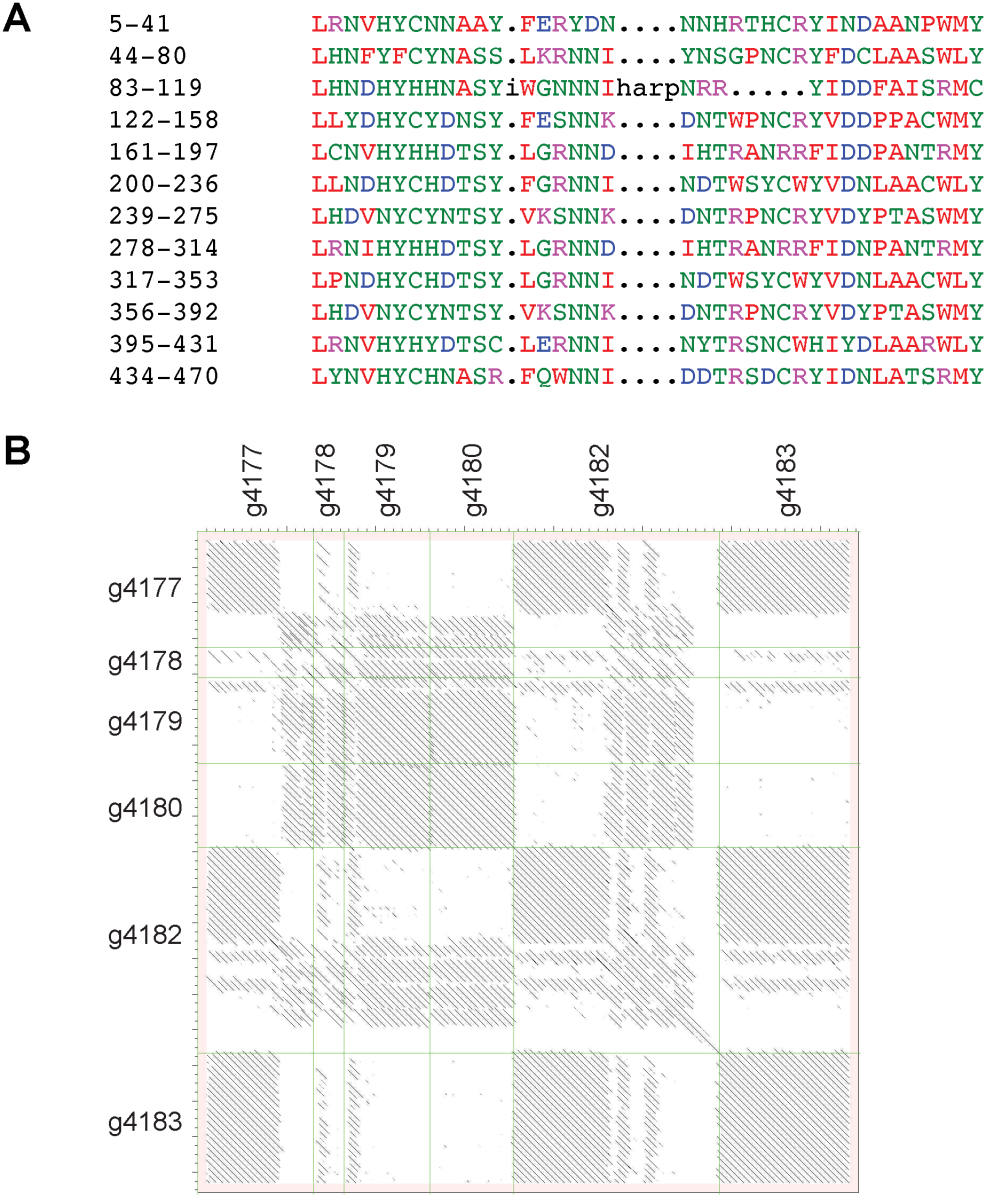
Unusual structure of *Drechmeria*-specific proteins and complex structural relationship between neighbouring proteins. (A) RADAR analysis [54] reveals the repeated structure in the sequence of g4180.t1, a 471 a.a. protein from OrthoMCL-defined paralogous group 2 (S6 Table). (B) All-against-all dot-plot representation [55] of the alignment of the predicted protein sequences from g4180.t1 and from 5 neighbouring genes on scaffold omap6908, all from the OrthoMCL paralogous group 2. Dots represent regions of sequence similarity (within a 100 a.a. sliding window). The intensity of each dot is proportional to the corresponding alignment score. The “.t1” suffix has been removed from all sequence names.

To define gene families and assign proteins to orthologous and paralogous groups, we performed an OrthoMCL analysis [52]. Most proteins (6851) were assigned to an OrthoMCL orthologous group, together with 241 for which an orthologue but no group was assigned (in total 81.2%; S6 Table). For the great majority (82%) of proteins allocated to an OrthoMCL orthologous group, the closest homolog was in *Fusarium graminearum* (the anamorph name of *Gibberella zeae*), the only Hypocrealean fungal species represented in OrthoMCL (S6 Table). The most populated groups, with 20 members (OrthoMCL group OG5_126718) correspond to predicted nonribosomal peptide synthases (see below), followed by ABC transporters and subtilisin-like serine proteases (OG5_134254 and OG5_137388, respectively, with 15 members each). When no homolog is found in any of the 150 species in OrthoMCL, proteins are placed into paralogous groups on the basis of their sequence [52]. The largest group of paralogs, with 15 members, corresponds to the heat-labile enterotoxin alpha chain, present in several insect pathogenic fungal species including *B*. *bassiana* and *Metarhizium robertsii* (e.g. EXU96489) [53]. A further 3 groups (containing a total of 18 predicted proteins) exhibited a more-or-less strong similarity to the heat-labile enterotoxin alpha chain, and one group of 5 to subtilisin-like serine proteases, also mentioned above (Tables 5 and S6). As discussed below, these all potentially play a role in fungal virulence or in the interaction of *D*. *coniospora* with other microbes. This analysis also revealed many groups of proteins currently unique to *D*. *coniospora*. In the most dramatic example, a group of proteins (OrthoMCL paralogous group 2) with no recognisable domain, each entirely composed of highly repeated sequences, has expanded to 10 members, with the majority found in a cluster of less than 50 kb on scaffold omap6908, with a very complex pattern of conservation (S6 Table and Figs 3 and S1). A further 9 out of the 20 groups with at least 4 members currently correspond to proteins unique to *D*. *coniospora* (Table 5).

**Table 5:** Families of predicted *D*. *coniospora* paralogs.

OrthoMCL group(s)*	Number of members	Identity
1, 4, 9, 17	15, 8, 6, 4	Heat-labile enterotoxin alpha chain
2, 5, 6, 7, 8, 10, 14, 15, 16, 19	10, 8, 7, 7, 6, 6, 4, 4, 4, 4	*Drechmeria*-specific; unknown function
3, 12, 13, 18, 20	9, 5, 4, 4, 4	Hypothetical protein conserved in certain fungal species; unknown function
11	5	Subtilisin-like serine protease

*See S6 Table

### Comparative analysis of protein domains

**Fig 4:**
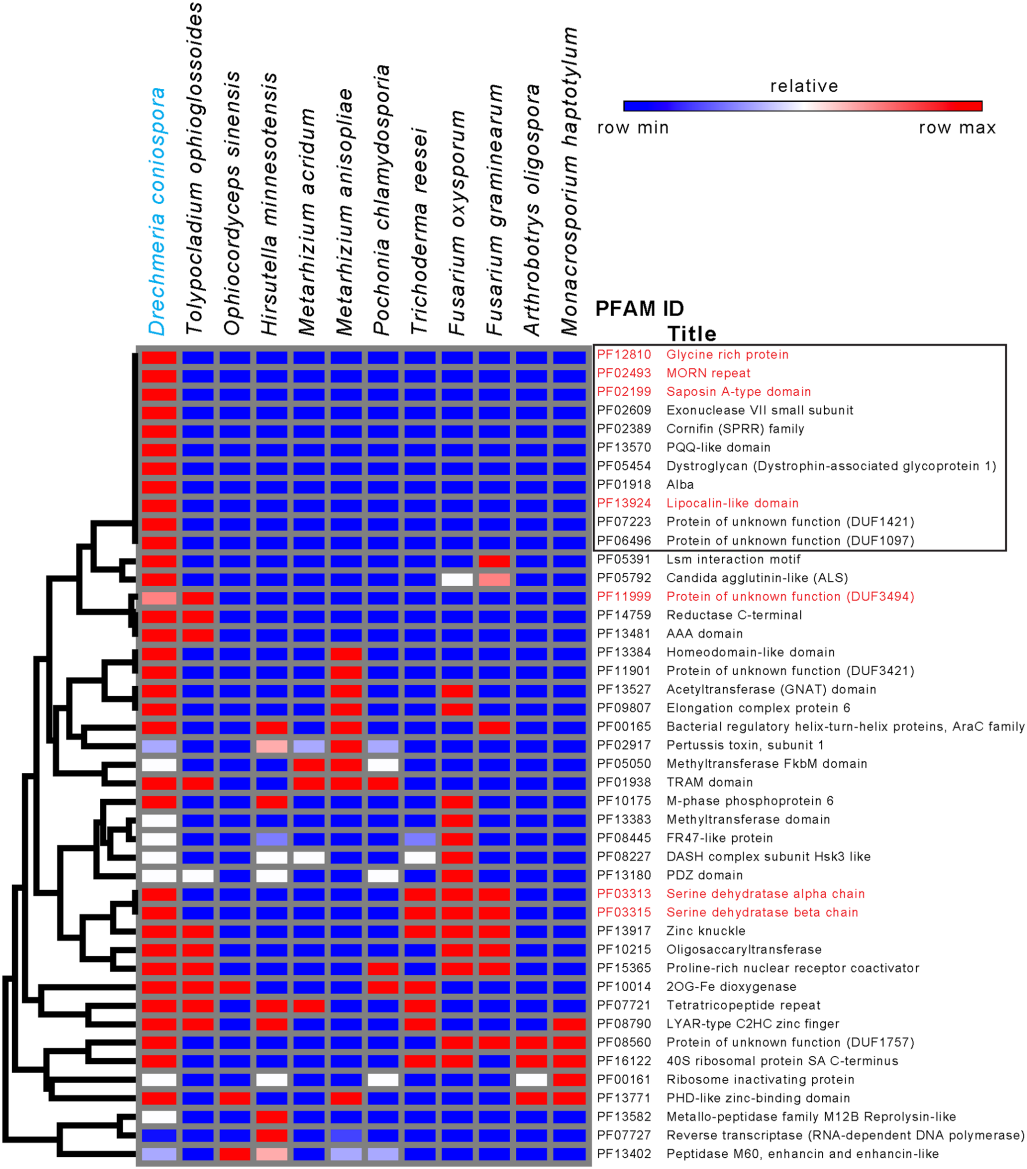
Species-specific or atypical protein domain families in the predicted *D*. *coniospora* proteome. Hierarchical clustering of protein domain families present in *D*. *coniospora* and not more than 4 of the other fungi, on the basis of the corresponding number of proteins. PFAM domains discussed in the text are highlighted in red. The box highlights 11 families specific to *D*. *coniospora* (see S1 Text). The colour code reflects the relative abundance of proteins with each domain, from high (red) to low (blue) across the different species. The serine dehydratase alpha and beta domains (PF03313 and PF03315, respectively), cluster since they occur in a single highly conserved protein (g4699.t1 in *D*. *coniospora*).

To look in more detail at protein domains, we next compared the PFAM annotations [56] for the entire set of predicted *D*. *coniospora* proteins with those of the 11 other fungi obtained with InterproScan using the same set of parameters (S7 Table). We completed the analyses of specific proteins and protein families by manual inspection. A total of 4287 PFAM domains were identified in at least one protein from one or more of the species, with 3510 (81.9%) represented in predicted *D*. *coniospora* proteins and more than half (55.7%) in all 12 predicted proteomes. These presumably reflect core eukaryotic and/or fungal biological processes. In the expectation of revealing domains that were potentially functionally related, we hierarchically clustered protein domain families present in *D*. *coniospora* and not more than 4 of the other fungi, but no obvious associations were found (Fig 4). A number of PFAM domains were predicted for *D*. *coniospora* proteins but not for proteins of any of the other 11 fungi (Fig 4 and S7 Table and S1 Text). Within this group, each PFAM domain is present in a single protein, with 3 exceptions. Three predicted *D*. *coniospora* proteins (OrthoMCL paralogous group 33, S6 Table and S1 Text) contain PF12810, the “glycine rich protein” domain, characterised by several glycine rich motifs interspersed through the sequence. Currently, no orthologues have been described in any other species, and no hint of a function can be garnered from the sequence. Two lipid-binding MORN (Membrane Occupation and Recognition Nexus; PF02493) domains [57] are predicted, towards the C-terminus, in proteins from each of 2 adjacent *D*. *coniospora* genes (g5037.t1 and g5038.t1; OrthoMCL orthologous group OG5_154358). MORN domains are relatively uncommon in fungi, but tandemly arranged orthologues for these 2 proteins do exist in one species, *Trichoderma gamsii*, and orthologs are currently also found in various other fungi, such as the brown-rot Basidiomycota *Hydnomerulius pinastri*. The conserved N-terminal portion of these proteins is shared with a number of related toxins, including the hemolytic factor neoverrucotoxin from stonefish venom (S5 Table). Whether these proteins may play a role in fungal virulence and interactions with other microorganisms is a matter for speculation. The presence of the Saposin A domain (PF02199) in 2 predicted proteins (g3895.t1 and g1982.t1, with 3 and 2 domains respectively) is equally atypical (S5 and S7 Tables). We address its possible role below.

On the basis of their constituent domains, several other atypical or highly represented protein families (Fig 4 and S7 Table) are also potentially linked to virulence. These include the single iron-sequestrating lipocalin (PF13924) and the deuterolysin M35 metalloprotease (PF02102; called here M35) domains. The M35 domain is unusually highly represented compared to other fungi [58, 59], being present in 10 predicted *D*. *coniospora* proteins.

Of a total of 777 domains absent from *D*. *coniospora*, 32 were present in all 11 other species (S7 Table). As a most striking example, *D*. *coniospora* lacks proteins containing the NACHT domain (PF05729), which is present between 8 and 117 times in the other species, suggesting that this is unlikely to simply be a problem of sequence coverage or gene prediction. In ascomycete fungi, the NACHT domain can be found together with the HET domain (PF06985) in heterokaryon incompatibility proteins. It acts as a death effector domain, preventing viable heterokaryotic cells from being formed by the fusion of filaments from different wild-type strains [60]. In *D*. *coniospora*, of the 6 proteins predicted to contain a HET domain, 5 (g3856.t1, g5845.t1, g5969.t1, g6809.t1, g7038.t1) do not contain any other domains, while in common with many other fungal proteins of this class, one (g215.t1) is coupled to an ankyrin repeat (PF12796) domain. No sexual stage has been described for *D*. *coniospora*, and in common with 3 of the 4 other nematophagous fungi, no mating type protein (MATα, PF04769) domain was predicted (S7 Table), making it unlikely that these different proteins play any role in heterokaryon incompatibility.

To see whether there was any general pattern for the 32 domains absent from *D*. *coniospora* suggestive of a coordinate evolutionary process, we used dcGO analysis to look for ontology term enrichment at the domain level [61]. This revealed a potential involvement of the NACHT domain together with 4 others (PF00931; PF01966; PF02178; PF09273) in the biological process, “regulation of response to stress” and of 4 of them (PF00931; PF01966; PF05729; PF09273) in “regulation of defense response”. This is discussed further below. Submitting all 777 domains absent from *D*. *coniospora* to dcGO analysis highlighted the absence of 16 related domains, all annotated as being involved in hydrolase activity, acting on glycosyl bonds, including PF00331 that corresponds to the Carbohydrate-Active Enzyme (CAZyme) glycosyl hydrolase (GH) family 10. There was also a significant (p = 2.5x10^-4^) enrichment for the more specific ontology term “alpha-N-arabinofuranosidase activity” (PF05270, PF06964, PF09206), suggesting an alteration in the capacity of *D*. *coniospora* to metabolise different carbohydrates compared to other fungi analysed.

### Carbohydrate-active enzymes

**Fig 5:**
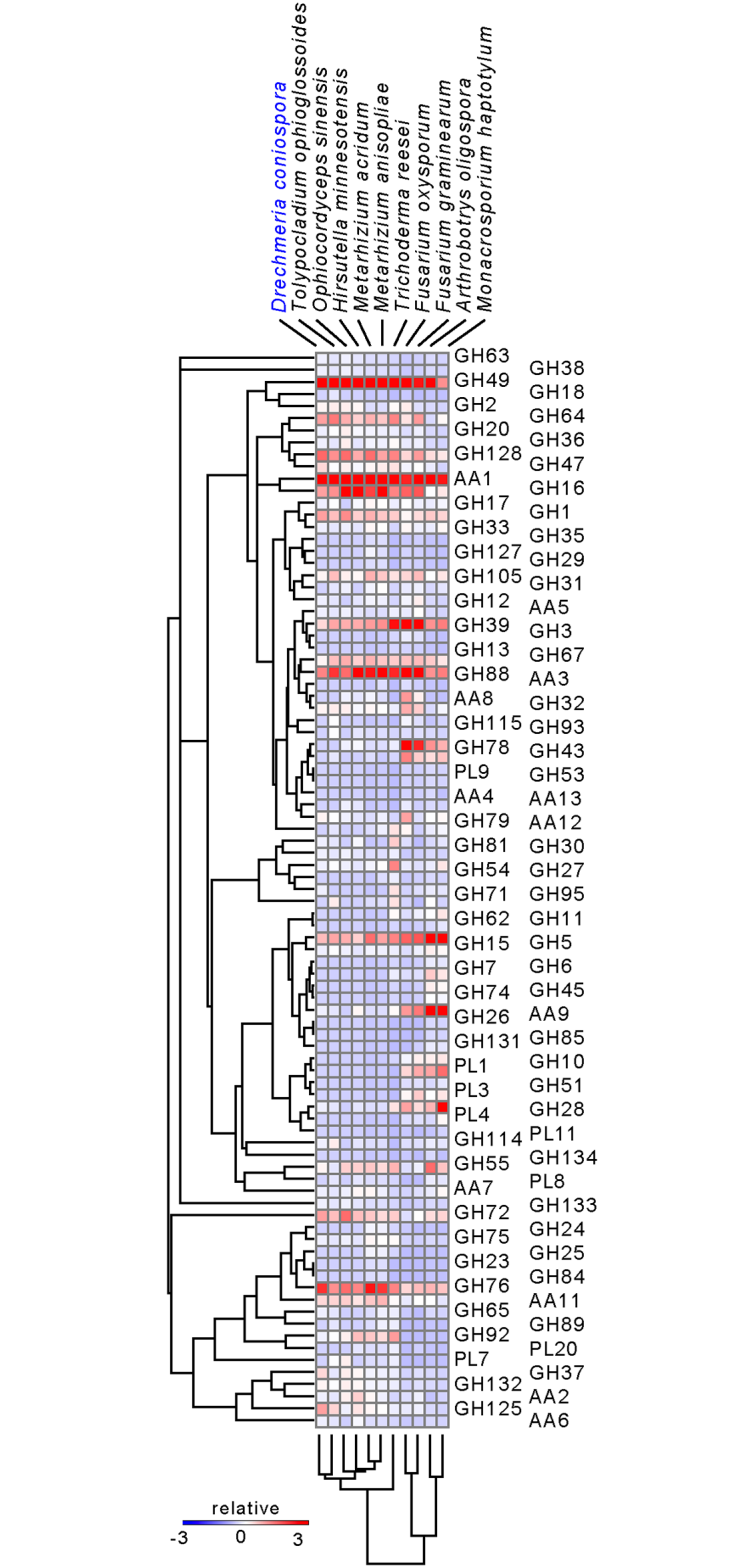
Supervised clustering of selected CAZy families in 11 fungal species. The distribution of the CAZy families involved in complex carbohydrate breakdown (AA, GH and PL classes) across the given species is shown. Clustering of families is based on the number of genes in each family. The colour code reflects the relative abundance of proteins within each family, from high (red) to low (blue) across an individual species.

A species’ set of CAZymes can often give insights into its biology, in particular into nutrient sensing and acquisition. Given the differences revealed by the dcGO analysis, we conducted a targeted examination of CAZymes in *D*. *coniospora* and 10 of the 11 fungi chosen for the other comparative analyses (S8 Table). *P*. *chlamydiosporia* was not included as it will be the subject of a dedicated study. Overall, CAZyme profiling recapitulated the phylogenetic analysis, except that the two *Metarhizium* species clustered together with *H*. *minnesotensis* and *O*. *sinensis* (Fig 5). This grouping of the various fungi reflects their respective requirements for carbon acquisition. The two nematode-trapping fungi *A*. *oligospora* and *M*. *haptotylum* have a large repertoire of enzymes for feeding on plant cell wall polysaccharides. They make a separate group and are neighbours of the saprophytes, reflecting their dual parasitic and saprophytic lifestyles. *T*. *reesei* has an intermediate position consistent with its evolving from an ancestral saprophyte lifestyle to become a mycoparasite. The remaining fungi, which are the most specialized and have evolved by gene loss, group together by virtue of their common loss of an arsenal of plant polysaccharide degradative enzymes. Thus the nematophagous and insectivorous fungi in this group are not separate from the mycoparasite *T*. *ophioglossoides*; the same range of CAZymes is probably needed for the three types of substrate and this is accompanied by a similar loss of the plant-targeting CAZymes. Regarding *D*. *coniospora* in detail, it has lost virtually all enzymes, from multiple families, that participate in cellulose binding (Carbohydrate Binding Module, CBM1), the breakdown of cellulose/hemicellulose and pectin-rich plant cell walls (e.g. GH7, GH45, PL8, and CE8 family proteins; S8 Table). The few GH5 proteins that remain in *D*. *coniospora* are predicted to be involved in the metabolism of fungal cell wall ß-glucans, not the digestion of plant cellulose or mannan. The GH13 family, involved in both starch and glycogen breakdown, has also shrunk to just two members. The two remaining proteins show strong similarities to glycogen branching and debranching enzymes and are thus most likely involved in the fungal glycogen cycle. Collectively, and coupled with the expansion of protease families and the acquisition of virulence factors, these changes appear to reflect the shift of *D*. *coniospora* to an obligate nematophagous lifestyle.

### Secondary metabolite biosynthesis

Another hallmark of each fungal species is its complement of genes involved in the production of secondary metabolites. The “backbone” genes for these biosynthetic pathways include those encoding nonribosomal peptide synthases (NRPSs), polyketide synthases (PKSs), and prenyltransferases (DMATSs), responsible for the production of bioactive peptides, polyketides, and indole alkaloids, respectively. SMURF analysis [62] revealed that the number and type of backbone genes predicted for *D*. *coniospora* was comparable to those of other Hypocreales fungi (Table 6). The majority of the NRPS and NRPS-like genes belong to OrthoMCL group OG5_126718. Several of the genes (g1025.t1 and g1029.t1; g3636.t1 and g3638.t1; g7201.t1, g7202.t1 and g7204.t1; g8001.t1 and g8002.t1) are close together in the genome suggesting that they may be functionally related (S9 Table). The molecules synthesized by NRPSs, PKSs and DMATS are frequently modified by “decorating” enzymes before secretion. The genes encoding the proteins necessary for these different steps are often found in genomic clusters, and are co-ordinately regulated by specific Zn_2_Cys_6_ transcription factors and/or by the global secondary metabolism regulator LaeA [62]. Of the 29 backbone genes, 24 had an associated gene cluster, and of these, 4 included a Zn_2_Cys_6_ gene. These are therefore candidate regulators of their respective clusters. Two clusters (14 and 15) included genes in the proximity of the *D*. *coniospora* LaeA-encoding gene g6733.t1 (S9 Table). In *D*. *coniospora*, LaeA could play a conserved role in secondary metabolism. It is important to note that in many fungal pathogens, secondary metabolites are essential for virulence [63].

**Table 6:** Number of secondary metabolite backbone genes predicted from the *D*. *coniospora* genome compared to other species.

	DMAT	NRPS	NRPS-Like	PKS	PKS-Like	Reference
***Drechmeria coniospora***	1	10	9	7	2	This study
***Tolypocladium ophioglossoides***	0	14	6	16	2	[64]
***Trichoderma reesei***	0	8	5	11	1	[62]
***Hirsutella minnesotensis***	6	21	21	27	4	This study
***Fusarium oxysporum***	2	7	12	9	2	[62]
***Fusarium graminearum***	0	10	11	14	1	[62]

### PHI-base analysis

To gain a general view of proteins potentially involved in virulence, we made use of the pathogen-host interactions database, PHI-base (phi-base.org; [65]). Of the 2104 proteins matching a PHI-base entry, 990 had the annotation “reduced virulence” or “loss of pathogenicity”, indicating that a homologous protein in at least one other species plays a demonstrated role as a virulence factor in a particular model of infection (S5 Table).

Among the highly represented (≥5) hits in PHI-base, several were characterised by the presence of ABC transporter domains, and so predicted to be involved in ATP-dependent export of organic anions or drugs from the cytoplasm (S10 Table and Fig 1). For predicted NRPS proteins, 9/20 members of the orthoMCL group OG5_126718 were assigned a PHI-base annotation (PHI:2511). The analysis also highlighted the potential role of multiple degradative enzymes, 11 chitinases (GH18; PHI:144; 6/6 OG5_126929, 2/2 OG5_142806, 2/2 OG5_210539, 1/1 OG5_152762), known to be important for the virulence of nematophagous fungi [7, 66], and subtilisin-like and extracellular metalloproteases (PHI:2117 and 479, with 9 and 5 members, respectively). These are often found in expanded gene families in pathogenic fungi (e.g. [67]). There were also 11 Pth11-like receptors (PHI:404), which can be involved in host sensing and have established roles in virulence in other species [68]. Three of them contain a CFEM domain (PF05730), found in proteins with proposed roles in fungal pathogenesis [69]. As expected from the OrthoMCL analysis, there were also multiple hits to enterotoxin A proteins (PHI:698, with the PF01375 domain), scattered throughout the genome (g496.t1, g964.t1, g2819.t1, g5058.t1, g6833.t1, g7169.t1, g7949.t1). The *D*. *coniospora* genome is therefore predicted to encode a large range of virulence factors, some of which have expanded markedly in number compared to other fungal species.

### *D*. *coniospora* secretome

**Fig 6:**
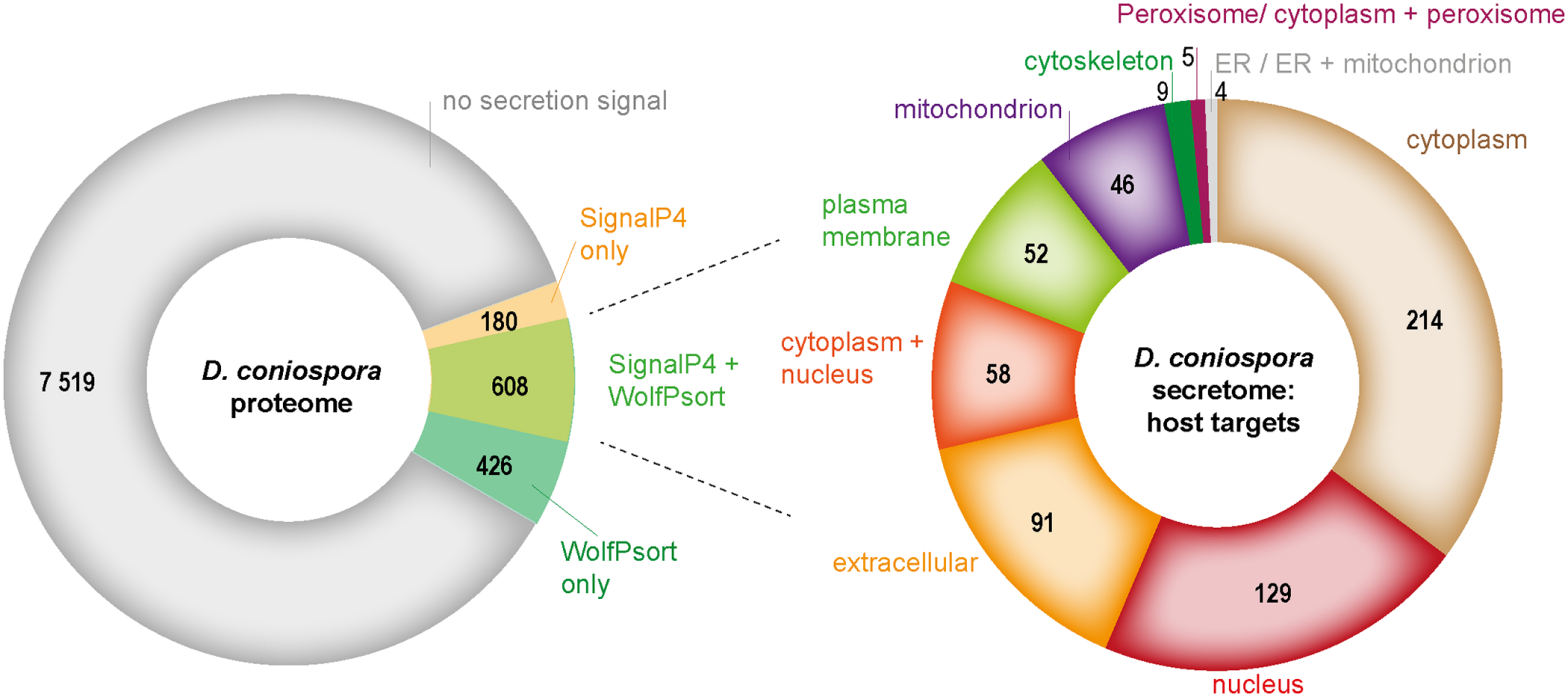
Predicted secreted proteins in *D*. *coniospora*. The left hand chart shows the distribution of protein predicted to be secreted by 2 different computational methods. For the proteins predicted to be secreted by both, the right hand chart indicates the predicted sub-cellular localisation.

To be able to act as virulence factors, many proteins, for example chitinases and proteases, need to be secreted. Some virulence factors are secreted into host cells, and can be targeted to specific organelles. We therefore complemented the InterproScan and PHI-base analysis with a focused and more thorough *in silico* investigation of the *D*. *coniospora* secretome. A total of 608 proteins (7%) were predicted with high confidence to be secreted (Fig 6 and S11 Table). They included the Saposin A domain protein g3895.t1, as well as 6/10 M35 domain proteins. More than a third of the putative secreted proteins (242/608), including 5 of the 6 secreted M35 domain proteins, and multiple proteins containing several different glycosyl hydrolase domains (GH2, 3, 16, 18, 20, 31, 35 47 and 65; see above), were also predicted to target a host cell organelle (e.g. nucleus or mitochondria), and of these 27 were homologous to proteins present in PHI-base with a demonstrated role in virulence (Fig 6 and Tables 7 and S11). In addition to chitinases, among the 27 predicted proteins, there were alkaline, aspartic, metallo-, subtilisin-like and cuticle-degrading proteases, all of which potentially contribute to the destruction of host tissue. There were also heat-labile enterotoxin homologues that would similarly be predicted to be direct effectors of virulence or play a role in *D*. *coniospora*’s interactions with other microbes (see below).

**Table 7:** Potential virulence factors among *D*. *coniospora* proteins predicted to be secreted and targeted to a host organelle.

Gene ID(s)	PHIBASE ID	BLASTP hit	Brief description
g3389.t1	PHI:434	GEL1_ASPFC	1,3-beta-glucanosyltransferase
g6911.t1	PHI:2117	ORYZ_ASPCL	Alkaline protease
g1715.t1	PHI:73	YPS3_YEAST	Aspartic proteinase
g2067.t1	PHI:1046	SOL5_ALTSO	Bifunctional solanapyrone synthase
g2448.t1	PHI:383	SOD5_ARTBC	Cell surface Cu-only superoxide dismutase
g375.t1, g3187.t1	PHI:144	CHI1_APHAL	Chitinase 1
g2033.t1	PHI:2117	CUDP_METAN	Cuticle-degrading protease
g3550.t1	PHI:2570	CYB2_WICAO	Cytochrome b2, mitochondria
g858.t1	PHI:3078	E9DYG9_METAQ	Endonuclease/exonuclease/phosphatase family protein
g656.t1, g6818.t1	PHI:479	MEP1_COCP7	Extracellular metalloprotease
g960.t1	PHI:1071	GLU2A_SCHPO	Glucosidase 2 subunit alpha
g964.t1	PHI:698	E2AA_ECOLX	Heat-labile enterotoxin IIA
g496.t1	PHI:698	E2BA_ECOLX	Heat-labile enterotoxin IIB
g1983.t1	PHI:2920	ASO_CUCPM	L-ascorbate oxidase
g2355.t1, g5020.t1	PHI:785	MU157_SCHPO	Meiotically up-regulated gene 157 protein
g6255.t1	PHI:184	PRY2_YEAST	Pathogenesis-related protein 2
g4517.t1	PHI:1071	AGDC_ASPFU	Probable alpha/beta-glucosidase agdC
g1115.t1	PHI:68	OPSB_ASPOR	Probable aspartic-type endopeptidase
g2153.t1	PHI:184	PRY1_ARTBC	Probable pathogenesis-related protein
g5968.t1, g6831.t1	PHI:2654	A2965_ARTBC	Putative amidase
g1191.t1	PHI:1166	ATG15_CHAGB	Putative lipase (Autophagy-related protein 15)
g4757.t1	PHI:2117	SUB2_PSED2	Subtilisin-like protease 2
g7479.t1	PHI:891	G4MVB6_MAGO7	Uncharacterized protein

### Comparative analysis of nematode-destroying fungi

**Fig 7:**
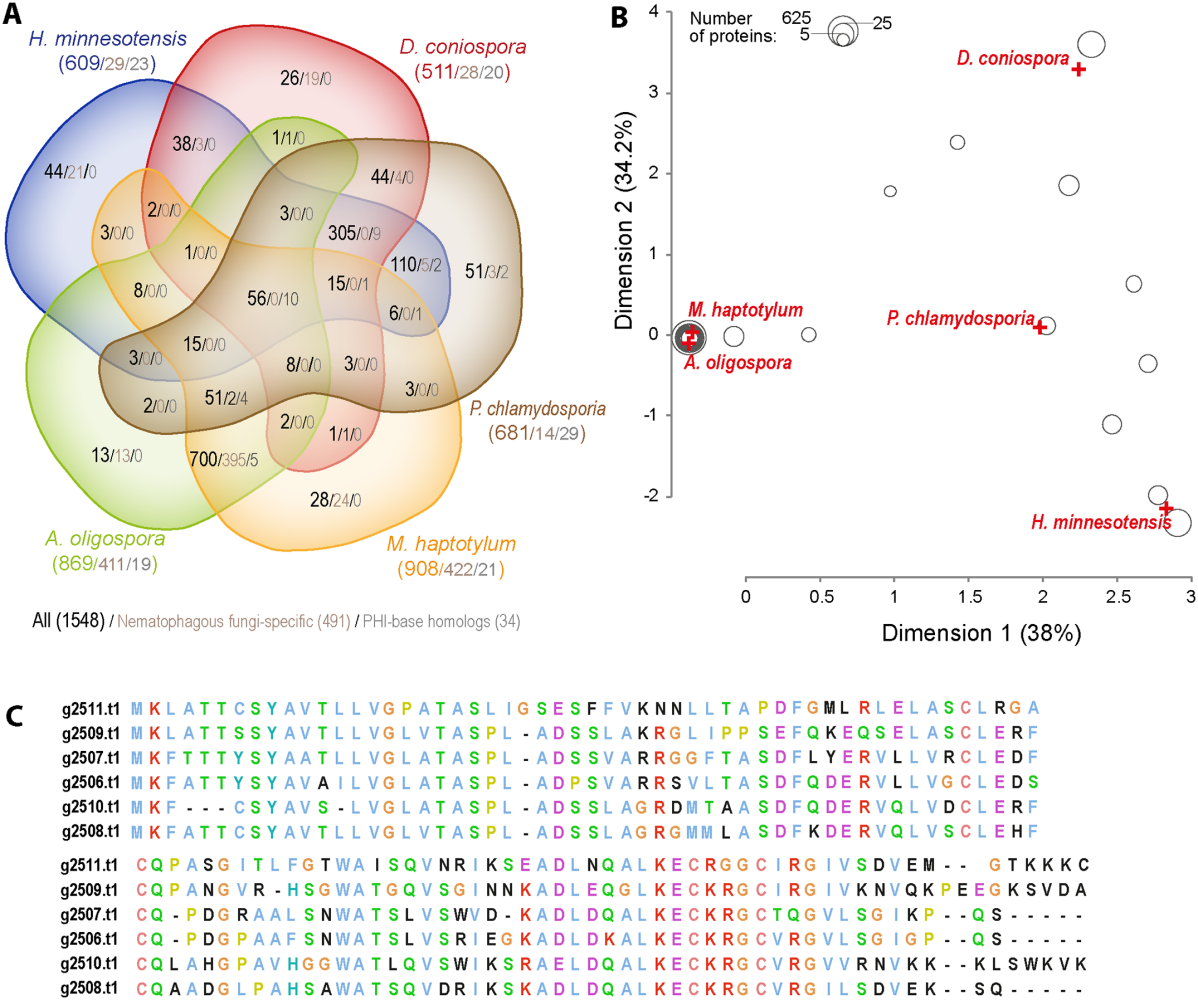
Comparative analysis of the predicted secretome of *D*. *coniospora*. (A) Distribution of sequence-based protein clusters across five nematopathogenic fungal genomes. Only clusters containing at least one secreted protein are shown. Except for empty sectors, in each sector, there are 3 numbers: total number of clusters/number of clusters with members only in nematophagous fungi/number of clusters with at least one member that matches a PHI base entry with an annotation of reduced virulence or loss of pathogenicity. (B) A correspondence analysis of sequence-based clusters of secreted proteins from five nematopathogenic fungi. The first two dimensions are shown. Crosses represent the position of the individual fungal species and circles represent protein clusters. Circles are sized according the number of constituent proteins as indicated. When clusters have identical coordinates, the size of the circle represents the sum of the number of proteins in each cluster. For example, the circle at (2.33, 3.6) corresponds to 19 clusters of proteins, in this case unique to *D*. *coniospora*, including Cluster01087. The proximity of each circle to the species’ apices is a measure of the contribution of the species to that cluster’s content. The distance between the circles is a measure of the similarity of their content (number of proteins from each species). (C) Multiple sequence alignment of proteins from the *D*. *coniospora*-specific cluster Cluster01087. Only 3 of the 6 proteins are predicted to be secreted (g2506.t1, g2508.t1, g2511.t1; S11 Table).

The above results illustrate how secreted proteins can be key to virulence. To investigate commonalities and differences in the molecular basis of nematode infection, we therefore conducted a comparative analysis of predicted secretomes between the 12 fungal species, focusing on the 5 that are nematopathogenic. Using reciprocal BLASTP analyses, we first determined high-confidence clusters of orthologous proteins among the 12 species. We then concentrated on the 1548 clusters containing at least one protein from a nematopathogenic species (S11 Table), and calculated their distribution across the 5 species (Fig 7A). While there was a substantial overlap between *A*. *oligospora* and *M*. *haptotylum*, with 395/700 shared clusters unique to these 2 species, very few of the clusters uniquely shared between *D*. *coniospora* and *H*. *minnesotensis* or *P*. *chlamydosporia* were restricted to nematopathogenic species (3/38 and 4/44, respectively). Indeed, there were only 9 clusters present in *D*. *coniospora* and another nematopathogenic fungus but not any of the 7 non-nematopathogenic species. Only one of these clusters corresponded to proteins with a conserved domain, namely fungal hydrophobin (PF01185), also found in rodlet proteins, a major component the hydrophobic sheath, or rodlet layer, that covers the surface of fungal spores, required in *Aspergillus nidulans* for efficient spore dispersal [70].

To gain a more synthetic and rigorous overview of these results, we used correspondence analysis of the clusters of the secreted proteins from the 5 nematopathogenic fungi. Correspondence analysis is conceptually similar to principal component analysis, but can be used with categorical rather than continuous data. The first 2 dimensions explained more than 70% of the observed distribution and separated the species. As expected from the numerical overlaps of protein clusters, *A*. *oligospora* and *M*. *haptotylum* were much closer together, and furthest from *D*. *coniospora* and *H*. *minnesotensis* (Fig 7B). The separation of the latter 2 species reflects their unique clusters. For *D*. *coniospora*, the most populated cluster of proteins (Cluster01087) contains 6 members (Fig 7C). The corresponding genes (g2506.t1-g2511.t1) are together in the genome. While the 6 proteins have no annotation or predicted function (S5 Table), 5 other clusters include at least one member with a PFAM domain. Consistent with our previous domain-centric analyses, among them we found the M35 (PF02102) and heat-labile enterotoxin alpha chain (PF01375) domains, reinforcing the notion that these domains potentially characterize the pathogenic capacity of *D*. *coniospora*. The remaining 3 were a domain of unknown function (PF11999), and 2 expected to be involved in fungal adhesion, the GLEYA (PF10528) domain, [71], and the hydrophobic surface binding protein A (PF12296) domain discussed in the next section. It is noteworthy that GLEYA domain proteins are highly expressed by nematode-trapping fungi during infection of *C*. *briggsae* [5].

### Stage-specific gene expression

While analysis of the genome sequence reveals predicted proteins putatively involved in virulence or other aspects of fungal physiology, it is necessary to establish when the corresponding genes are in fact expressed. To gain a first insight into the genes potentially important at different stages of the life cycle of *D*. *coniospora*, we chose to compare gene expression between mycelia that had been grown in liquid for several generations in the absence of nematodes, and spores harvested from infected *C*. *elegans* and starting to germinate *in vitro* (S2 Fig). These two morphological forms were expected to provide a broad though not necessarily exhaustive repertoire of expressed transcripts. The combined set of 48.6 million paired-end reads were mapped to the set of predicted genes. Inspection of the most highly expressed genes (arbitrarily the top 50) in mycelia and spores, revealed a substantial number of genes encoding basic metabolic enzymes, such as glyceraldehyde 3-phosphate dehydrogenase, as well as multiple proteins involved in translation (ribosomal proteins and elongation factors) and protein folding (chaperones of the heat shock protein family), presumably reflecting the need for protein synthesis during growth. We then used stringent criteria to define a small set of genes that were differentially regulated between germinating spores and mycelia, (61 and 86 genes, respectively; S12 Table). The list of spore-specific genes was particularly interesting. It included 16 encoding predicted proteins with no identified PFAM domain or homolog in UniProtKB. Several are found in the secretome-associated and/or orthoMCL-defined clusters described above including g2508.t1 and g2509.t1 (group 6 and Cluster01087), g4179.t1 (group 2), and g2083.t1 (group 34). The functional role in spores of the members these different paralogous groups merits further investigation in the future.

Two genes g3607.t1 and g6474.t1 encode proteins that contain the hydrophobic surface binding protein A domain (PF12296) mentioned above. By analogy with the eponymous protein from *Aspergillus oryzae* [72], we hypothesize that this protein is important for spore attachment to the cuticle of *C*. *elegans* and that further, they may act together with g5675.t1 that contains a CFEM domain (PF05730 [69]), that is structurally related to fungal adhesins and is highly preferentially expressed in spores. The differential expression of such genes will depend on stage-specific expression of transcription factors, such as g5153.t1 that is predicted to be a transcription factor with Zn_2_Cys_6_ (PF00172) and fungal-specific (PF11951) domains, and is also preferentially expressed in spores (S12 Table).

Genes that are more highly expressed in mycelia than spores would be predicted to be important for vegetative growth but also potentially for virulence. The list of these genes included candidates in both categories (S12 Table). Thus, on the basis of the domains found in the corresponding predicted proteins, 5 genes are associated with carbohydrate metabolism (GO:0005975; g1835.t1, g1896.t1, g3555.t1, g7200.t1, g7950.t1), while g7950.t1 corresponds to a pyruvate/2-oxoglutarate dehydrogenase, a key metabolic enzyme, and fg7260.t1 to a sulfide:quinone oxidoreductase that catalyzes the first step in the mitochondrial metabolism of H_2_S. These are expected to fulfil metabolic needs that are not present during the early growth of spores.

Otherwise, there is g3889.t1 that encodes a highly conserved protein, annotated as a putative NRPS-like enzyme in multiple fungal species; it was not identified as such by SMURF analysis. g3999.t1 encodes a subtilisin and was also preferentially expressed in mycelia. Subtilisin-type proteases are associated with virulence, including nematicidal activity [73], in many species [6]. As in other nematophagous fungi [8], as detailed above, *D*. *coniospora* has a large family of subtilisin-type proteases (PF00082; S7 Table and Table 5). These different genes are all potentially linked to growth in the nematode host. Strikingly, 46 of the 86 (53.5%) proteins corresponding to genes preferentially expressed in mycelia currently have no identifiable domains nor homologs in the UniProtKB database, compared to 30.8% for the full set of 8733 predicted proteins (S5 and S12 Tables), and among them 41/46 are shorter than the median length. Manual searches suggest that many may have homologues in other fungal species. As a single example, fg6211.t1 is expressed at almost 10-fold higher levels in mycelia than spores, encodes a predicted 70 amino acid protein and matches a predicted 64 amino acid protein of unknown function from *Trichoderma reesei* (Genbank XP_006962079). Determining the role of these different genes will require extensive functional analyses in the future.

### Gene expression during infection of *C*. *elegans*

While this analysis revealed genes potentially involved in virulence, an important question is what fungal genes are actually expressed during infection. Having the annotated *D*. *coniospora* genome in hand allowed a re-examination of RNAseq data obtained from samples of *C*. *elegans* infected by *D*. *coniospora* (NCBI SRA SRX036882 and [74]). From sequencing of samples taken 5 and 12 h post-infection (p.i.), a small number of reads among those that did not align to the *C*. *elegans* genome could be aligned to predicted *D*. *coniospora* genes. Together, 537 gene models were covered by at least one read, with 339 only at 5 h (p.i), 142 only at 12 h (p.i.) and 56 at both time-points (S13 Table).

Focusing on genes for which there were at least 3 matching reads (S13 Table), as might be expected, many corresponded to genes that were highly expressed in mycelia and/or spores (30/47 within the top 15 percentile for expression; i.e. >1192 and >1298 reads, for mycelia and spores respectively; S12 Table). In addition to 14 ribosomal genes, they included the CFEM domain protein, g5675.t1, mentioned above. Six encode proteins homologous to ones present in PHI-base with a demonstrated role in virulence. For example, g6659.t1 corresponds to a component of the mitochondrial membrane ATP synthase complex (S13 Table). As with the other such genes, its role in virulence probably reflects a general function in fungal physiology and growth.

For the remaining 17 genes that were not highly expressed in mycelia and/or spores, BLASTP searches at NCBI lead to the identification of potential homologs for 15 of the corresponding predicted proteins (e-value <10^−10^), across a range of species (Table 8). Among them, 3 encoded homologues of PHI-base listed virulence factors (S5 Table and Table 7). The first, g2153.t1 (PHI:184), potentially encodes a cysteine-rich secretory protein family (PF00188) member, similar to the pathogen-related protein Pry1. Members of this family have different roles in various pathogenic fungal species, including the neutralization of host defenses, and antimicrobial activity to inhibit the growth of competing microorganisms [75]. The second, g2390.t1 (PHI:257), encodes a widely conserved cell surface protein of unknown function, and the third, g6908.t1 (PHI:2117), an alkaline serine protease, homologous to the peptidase S8 of *Beauveria bassiana*. Interestingly, among the 17 genes, there were 3 groups of neighbours, suggesting a coordination of gene expression at the genome level. These included a group of 3 genes, g4815.t1–g4817.t1, that encode proteins that are completely unrelated in sequence: g4815.t1, one of 3 *D*. *coniospora* GH128 proteins, from a recently described glycoside hydrolase family ([76]; S8 Table), g4816.t1, a G-protein coupled receptor, and g4817.t1 that does not currently have homologues in any other species. For the other predicted proteins, it is notable that 2 encode proteins with a hydrophobic surface binding protein A (PF12296) domain, also found in chitinases (e.g. KID89971). One of these, g6474.t1, was mentioned above since it is preferentially expressed in spores, and may be important for the initial adhesion to and penetration of the nematode cuticle. This is consistent with the fact that the corresponding RNAseq reads were only found at the early time-point of infection.

**Table 8:** Selection of *D*. *coniospora* genes expressed during the infection of *C*. *elegans*.

	Read counts					Best Genbank BLASTP hit			PFAM domains	
Gene ID	5 hour p.i.	12 hour p.i.	Mycelia	Spores	Length (a.a.)	.Genbank	Length (a.a)	Species	ID	Name
g2153.t1	8	0	81	189	307	GI:302895657	324	*Nectria haematococca*	PF00188	Cysteine-rich secretory protein family
**g2389.t1**	3	0	40	29	118	GI:799240667	128	*Hirsutella minnesotensis*	-	
**g2390.t1**	4	0	57	34	303	GI:667661323	308	*Beauveria bassiana*	PF11327	Protein of unknown function (DUF3129)
g261.t1	0	4	411	167	69	GI:952549466	69	*Aspergillus lentulus*	PF11034	Protein of unknown function (DUF2823)
g3607.t1	7	2	147	593	428	GI:908391879	373	*Tolypocladium ophioglossoides*	PF12296	Hydrophobic surface binding protein A
g4094.t1	10	11	26	73	111	-	-	-	-	
g4805.t1	3	0	123	82	323	GI:666406534	327	*Stachybotrys chartarum*	-	
**g4815.t1**	3	1	3	35	354	GI:908393692	353	*Tolypocladium ophioglossoides*	PF11790	Glycosyl hydrolase catalytic core
**g4816.t1**	3	2	0	54	343	GI:949387848	548	*Rosellinia necatrix*	PF12430	Abscisic acid G-protein coupled receptor
**g4817.t1**	4	0	3	262	446	-	-	-	-	
g5054.t1	3	0	23	111	363	GI:969886810	403	*Trichoderma gamsii*	-	
g5671.t1	1	5	422	198	163	GI:799246947	108	*Hirsutella minnesotensis*	-	
**g6474.t1**	10	0	40	426	331	GI:389629282	257	*Magnaporthe oryzae*	PF12296	Hydrophobic surface binding protein A
**g6475.t1**	3	0	89	166	600	GI:743663295	600	*Metarhizium guizhouense*	PF01532	Glycosyl hydrolase family 47
g6844.t1	2	1	77	159	160	GI:531863496	231	*Ophiocordyceps sinensis*	-	
g6908.t1	2	1	5	4	563	GI:667643613	395	*Beauveria bassiana*	PF00082/PF05922	Subtilase family/Peptidase inhibitor I9
g7182.t1	3	0	107	161	416	GI:672821344	241	*Mortierella verticillata*		

The Gene IDs in bold indicate genes that are neighbours in the genome. Those that are underlined are homologous to proteins in PHI-base annotated as being important for virulence.

### Transformation and genome modification

**Fig 8:**
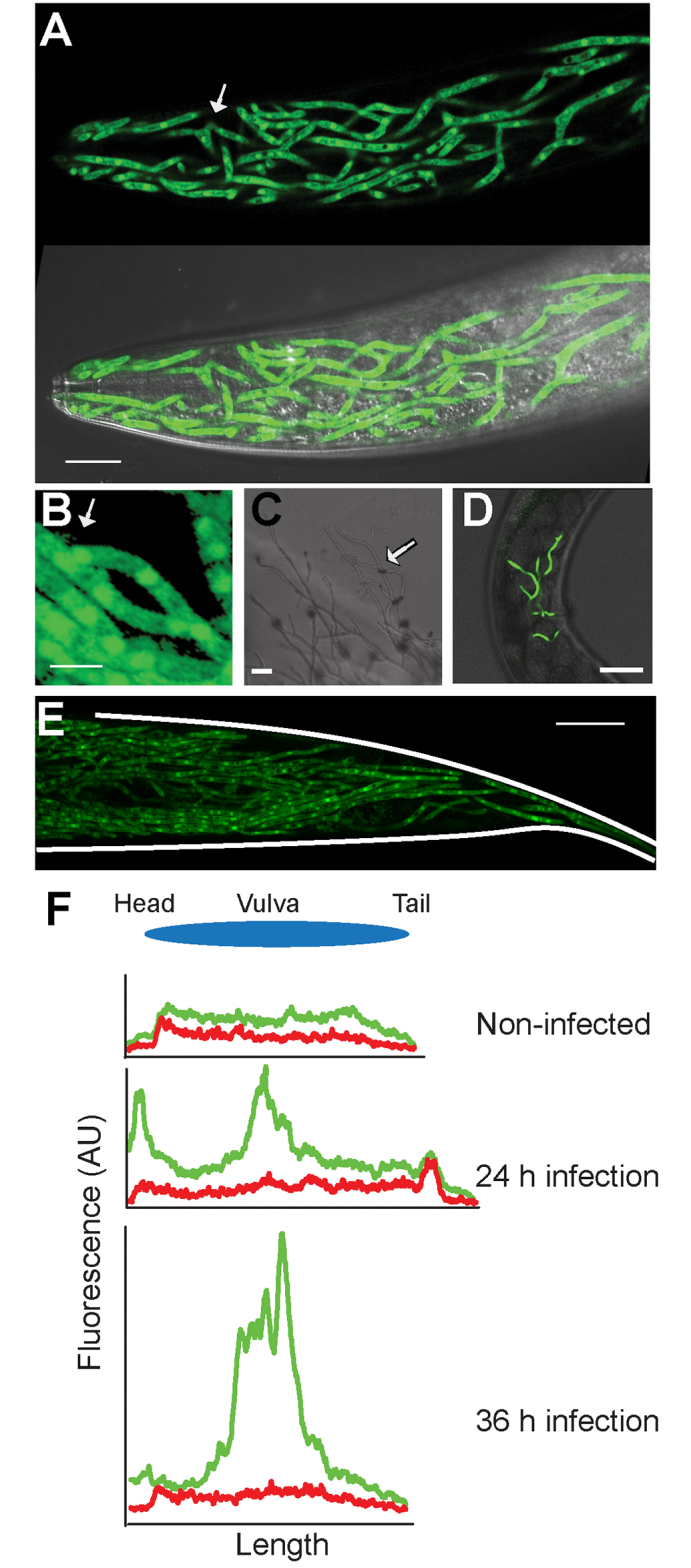
Transformation of *D*. *coniospora*. (A) A recombinant strain of *D*. *coniospora*, expressing GFP under the control of a ß-tubulin promoter, viewed by fluorescence microscopy (upper panel) combined with differential interference contrast microscopy (lower panel; scale bar, 20 μm). The fusion of 2 mycelia is highlighted by an arrow. (B) Higher magnification view of fused mycelia (scale bar, 5 μm). (C-E) The *Dso* mutant has a defect in anastomosis. (C) As highlighted with the arrow, in culture, mycelia are seen to grow across one another but never fuse. The mutant strain was engineered to express GFP constitutively. Fungal mycelia growing in living animals, visualized using a stereo fluorescence dissecting microscope (D), or confocal fluorescence microscope (E; the shape of the worm is traced by white lines), were not observed to fuse. Worms had been infected overnight (D) or for 60 h (A, B, E) before images were taken. Scale bars in C, D, E: 20, 50, 40 μm. (F) The growth of the fungus can also be followed using the Profiler of the COPAS Biosort. The graphs show fluorescence profiles for green and red channels for an uninfected worm (top); a worm infected at the head and vulva (peak in green signal on the left and in the middle, respectively in the middle graph) and analysed after 24 h; another worm infected at the vulva (peak in green signal in the middle, bottom graph) and analysed after 36 h. Fluorescence and length are measured in arbitrary but constant units.

The different *in silico* analyses reported above led to the identification of a very large number of candidate virulence genes. Addressing their functional importance would be greatly facilitated by the availability of techniques for the genetic transformation of *D*. *coniospora* and for targeted editing of its genome. By screening a number of different liquid media (C. Couillault, personal communication), we found that *D*. *coniospora* grew well in a rich, cholesterol-supplemented medium. We used fresh liquid cultures of *D*. *coniospora* mycelia to generate protoplasts, which were then transformed using a standard technique of polyethylene glycol (PEG)/CaCl_2_-mediated DNA uptake [77]. As a proof of principle, we transformed protoplasts with a plasmid (pLH4237) in which expression of a gene encoding a chimeric hygromycin B phosphotransferase::GFP protein [78] was driven by the *D*. *coniospora* ß-tubulin promoter (*ß-tub*p::HPH::GFP). The resultant recombinant fungus exhibited hygromycin resistance and strong GFP fluorescence in both spores and mycelia (Fig 8A).

To test the possibility of specifically knocking out a gene’s function, we chose to target the *D*. *coniospora* homolog of the *so* (*soft*) gene (NCBI Gene ID: 3880225), required in other fungi for anastomosis (mycelial fusion) since this was expected to give a clear viable and visible phenotype [79], while at the same time not greatly altering virulence [80]. We therefore flanked our *ß-tub*p::HPH::GFP construct with arms homologous to the 5’ and 3’ regions of the *D*. *coniospora so* gene (*Dso*; g1469.t1) and used this construct (in pLH4256) to transform protoplasts. We obtained hygromycin-resistant GFP-expressing transformants in which, as demonstrated by PCR (S3 Fig) and sequencing, the *Dso* gene was replaced by the *ß-tub*p:: HPH::GFP cassette. The mutant exhibited the expected anastomosis defect. In contrast to the wild-type strain (Fig 8A and 8B, S2B and S2C Fig), neighbouring mycelia were never observed to fuse *in vitro* (Fig 8C) or during infection of *C*. *elegans* (Fig 8D and 8E). We have therefore the capacity to make targeted modifications of the *D*. *coniospora* genome.

Having fungal strains that express a fluorescent protein opens many new possibilities for future research. One immediate consequence is that we were able to follow the infection *in vivo* directly using fluorescence microscopy (Fig 8A, 8B, 8D and 8E). We also wondered whether this would offer a new way to quantify the progression of the infection. Fluorescence in *C*. *elegans* can be measured *in vivo* using the Complex Object Parametric Analyzer and Sorter (COPAS) Biosort. When the Biosort’s Profiler is used, as well as a single measurement for each worm, one obtains a readout of fluorescence intensity along the length of the worm [81]. We found that the COPAS Biosort was sufficiently sensitive to allow us to follow the progression of the infection in a qualitative (Fig 8F) and potentially quantitative manner.

### Initial characterisation of a potential fungal counter-defensive strategy

Our *in silico* analysis highlighted the atypical presence of 2 proteins with saposin A (PF02199) domains, one of which (g3895.t1) was predicted to be secreted (S11 Table). Mammalian saposins are synthesized as precursor proteins (prosaposin) that contain four Saposin-B domains (PF05184; PF03489) and two Saposin-A domains that are removed during the process of activation. Saposin-B domains also occur in proteins without Saposin-A domains across many species including nematodes. In *C*. *elegans*, they are represented by a large family of 23 proteins, also called caenopores [82, 83]. They are structurally similar to the innate defense proteins of the SAPLIP family, including vertebrate NK-lysin and granulysin [84]. Several of them are upregulated upon infection, some by multiple pathogens ([85] reviewed in [22]), including *spp-2*, *spp-6*, *spp-13*, *spp-14* and *spp-15* that are induced upon infection by *D*. *coniospora* [74]. A number of the SPP caenopores/saposins have been demonstrated to play a role in host innate immunity [86, 87] suggesting that certain SPP proteins could be also be direct effectors of antifungal defense.

The *D*. *coniospora* protein g3895.t1 is characterized by the presence of 3 Saposin-A domains, but no Saposin-B domain. There are currently no clear orthologs in any species in publically available databases. Given its unusual structure, we hypothesised that this protein, which we call here SapA, might act as an inhibitor of one or more nematode Saposin-B domain-containing caenopores/saposins and thereby interfere with host defense.

**Fig 9:**
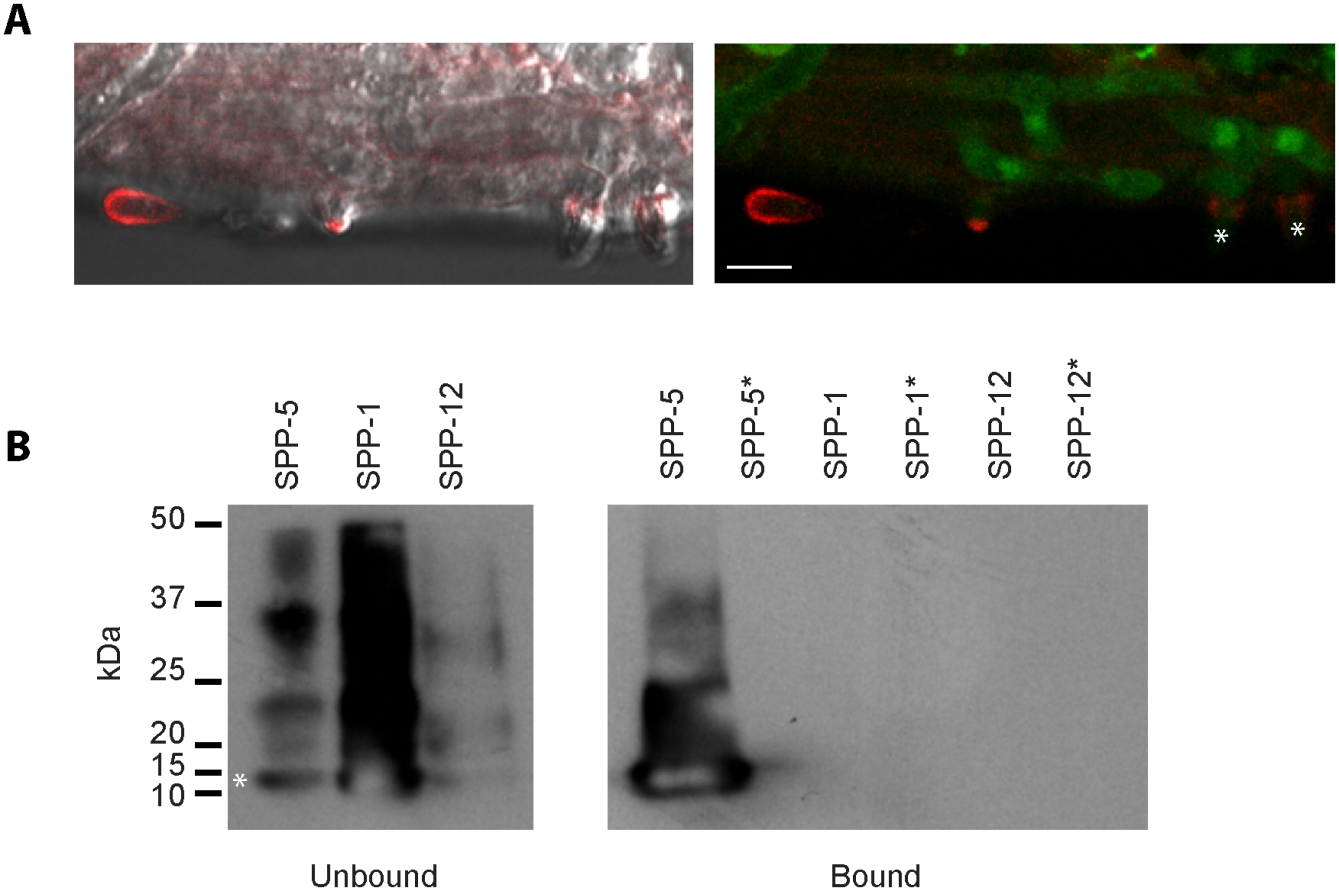
Saposin A-domain protein expression during infection of *C*. *elegans* and its *in vitro* interaction with SPP-5. (A) A strain of *D*. *coniospora* engineered to express GFP constitutively and a SapA::dsRed chimeric protein under the control of the *sapA* promoter, visualized using confocal fluorescence microscope (right panel) combined with differential interference contrast microscopy (left panel), during the infection of *C*. *elegans*. In both panels, a bright red fluorescent spore can be seen on the left. In the centre, a mycelium that is starting to exit the worm shows bright red fluorescence at its tip. On the right, 2 adjacent mycelia that have emerged can be seen. At the point where they leave the epidermis, a ring of SapA::dsRed can be seen, marked by asterisks in the right-hand panel. A general, less concentrated, signal can also be seen in the infected tissue. Worms had been infected for 60 h before images were taken. Scale bar = 5 μm. (B) Physical interaction between SPP-5 and SapA::dsRed. Recombinant His-tagged SPP-1, SPP-5 or SPP-12 was mixed with a protein extract from fungi expressing SapA::dsRed. The mix was analysed by Western blot probed with an anti-His-tag antibody before (left hand panel) or after (right hand panel) immunoprecipitation with anti-dsRed antibody-coated beads. In the sample before immunoprecipitation, in addition to the band at the expected size (11.9 kDa) marked by an asterisk, higher molecule weight species were detected, corresponding to the previously described oligomerization [86]. SPP-5 was co-immunoprecipitated with SapA::dsRed, principally in its monomeric form, but not if incubated with blocked beads (lanes marked with an asterisk; a control for non-specific binding). Neither SPP-1 nor SPP-12 gave any indication of co-immunoprecipitating with SapA::dsRed, even if SPP-1 was more abundant in the sample before immunoprecipitation.

As a first step in the analysis of *sapA*, we chose to assay directly its expression during the infection of *C*. *elegans*. Using the *D*. *coniospora* actin gene as a control, by RT-PCR we observed a clear increase in the relative level of expression of *sapA* across the time-course of infection (S4A Fig). To define *in vivo* the spatio-temporal expression pattern of the corresponding protein, we made use of our capacity for transformation to produce recombinant fungus expressing the SapA protein tagged with dsRed at its C-terminus (SapA::dsRed), under the control of its own promoter. Strong dsRed expression was observed at the surface of spores, but not on mycelia early in the infection. Expression was then seen at the tips of growing hyphae at the moment when they approached the apical surface of the epidermis, before penetrating the cuticle from the inside (Fig 9A).

To test the hypothesis that the *D*. *coniospora* SapA might interact physically with one or more of the *C*. *elegans* caenopores/saposins, we incubated an extract of proteins purified from the *D*. *coniospora* strain expressing SapA::dsRed with 3 different purified recombinant *C*. *elegans* SPP proteins [83, 87], each possessing a C-terminal His-tag. The SapA::dsRed was then immunoprecipitated, together with any bound SPP protein, using an anti-dsRed antibody. To probe for a possible interaction with the SPP proteins, the immunoprecipitated material was analysed by Western blotting, using an anti-His-tag antibody. Although the 3 samples had been incubated with equal quantities of fungal protein, while there was no indication of any interaction between SapA::dsRed and SPP-1 or SPP-12, we observed a clear co-immunoprecipition of SapA::dsRed and SPP-5 (Fig 9B). Interestingly, SPP-5 is markedly more closely related to SPP-2 (79% similar) and the other infection-induced SPPs than are either SPP-1 or SPP-12 (S4B Fig).

## Discussion

*D*. *coniospora* was first described 75 years ago as a parasite of *Rhabditis* nematodes in leaf mold, and then named *Meria coniospora* [88]. Its spores adhere to many different nematode species, and it is capable of infecting a relatively broad range of hosts including *C*. *elegans*, soybean cyst and root knot nematodes [14, 89–93]. While the different steps in the infectious process have been documented at the ultrastructural level [10, 94], nothing is known at the molecular level. Here, we have provided a first annotated genome sequence of *D*. *coniospora* that will serve as a starting point for future functional studies, as well as for more refined predictions of gene structure (S1 Text).

To make a comparative study of the nematophagous lifestyle, we chose 4 fungal species that use different strategies to infect nematodes. Like *D*. *coniospora*, *H*. *minnesotensis* is an endoparasitic fungus of the family Ophiocordycipitacea. It naturally infects soybean cyst nematodes using non-motile spores. Although it has never been reported to be one of its natural pathogens, it can infect *C*. *elegans* in the laboratory [9, 95]. *M*. *haptotylum* and *A*. *oligospora* are phylogenetically distant species that infect diverse nematodes, including *C*. *elegans*, after trapping them with their adhesive knobs and nets, respectively. *P*. *chlamydosporia* lies between these 2 pairs phylogenetically, infects nematode eggs, and in common with the nematode trapping fungi can grow as a saprophyte [3, 4, 6, 96–102].

Our phylogenetic analysis confirms the assignment of *D*. *coniospora* to the family Ophiocordycipitacea. It is interesting to note that other members of the Ophiocordycipitaceae family, *Haptocillium sphaerosporum* and *Harposporium spp*. are also pathogens of *C*. *elegans* [20, 74]. Among sequenced fungi, *D*. *coniospora*’s closest relative is currently the truffle-parasite *Tolypocladium ophioglossoides*. Our analysis thus provides a further illustration of the polyphyletic nature of nematode parasitism and will contribute to the on-going debate regarding the acquisition of host specificity in pathogenic fungi [6, 67].

One factor that can contribute to the emergence of virulence traits is the amplification of transposable elements that facilitate genome rearrangements. The *H*. *minnesotensis* genome represents an extreme example since 35% of its genome was reported to be composed of transposable elements [9]; in *D*. *coniospora* they make up less than 2%. *D*. *coniospora* also has a substantially reduced range of glycoside hydrolase (GH) enzymes compared to the nematode-trapping fungi, in direct contrast to the extensive repertoire of *P*. *chlamydiosporia* [98]. This presumably reflects a decreased capacity to adapt to diverse environments [103]. Unlike *P*. *chlamydiosporia* that can infect plants as well as nematodes, *D*. *coniospora* is an obligate nematode-specific parasite [104]. This slimming down of the genome is reflected in diverse other protein families. Thus, in addition to the different families described above, for example, there are few multi copper oxidases and lytic polysaccharide mono-oxygenases, so-called “auxiliary activities” linked to lignocellulose conversion [105]. Its genome also contains fewer genes related to sugar/inositol transport, which are involved in the establishment of plant-fungus relationships in *M*. *anisopliae* [106]. Thus *D*. *coniospora* does not show the pattern of gene family expansions observed in characterized nematode-trapping fungi, which are more similar to those seen in plant pathogens than to insect and animal pathogens [5].

An atypical loss from the *D*. *coniospora* genome is of genes encoding NACHT-domain proteins, which as mentioned above are present in multiple copies in all the 11 other fungal species analysed. The NACHT domain is a constituent domain in one of the two main classes of NOD domain proteins. The second class has the NB-ARC (PF00931) domain, a signalling motif shared by plant resistance gene products and regulators of cell death in animals [107]. These too are absent from the predicted set of *D*. *coniospora* proteins. Again this is highly unusual, since they are found often in all the other species, (8, 9, 10 and 15 times in *M*. *haptotylum*, *A*. *oligospora*, *P*. *chlamydosporia* and *H*. *minnesotensis*, respectively). Indeed, NOD domain proteins are present broadly among fungi and have important roles in fungal non-self recognition and in defence systems [60]. Determining the reasons and consequences of this intriguing loss of NOD domain proteins from *D*. *coniospora* remains a challenge for the future.

On the other hand, both insect and nematode parasitism requires a broad set of genes involved in detoxification and resistance to oxidative stress, such as glutathione S-transferases, cytochrome P450 genes, and carboxylesterases [40]. These are present in the *D*. *coniospora* genome in similar numbers to its entomopathogenic neighbors. More than 100 protein kinases were detected. Many are likely to regulate the infection process, as previously described, for example, for a *M*. *anisopliae* protein kinase A [108] for which there is a direct *D*. *coniospora* ortholog (g2806.t1). They may in turn be regulated by different families of transcription factors, including the Zn_2_Cys_6_ fungal-type, bZIP and bromodomain-containing families, which are all well-represented in the *D*. *coniospora* genome, and are often involved in the control of gene expression linked to virulence [109].

As previously observed for other nematophagous species, the specialization of *D*. *coniospora* is also reflected in the high degree of structural innovation in its predicted proteome. Thus, for example, in *H*. *minnesotensis*, 11% of proteins were described as species-specific and lacking any recognisable domain [9]. In *D*. *coniospora* the corresponding figure is currently 16.3%. With increased sampling of closely related species, these figures will drop in the future. Nevertheless, in addition to the many readily predictable virulence factors, there is a wealth of novel biology to be explored using *D*. *coniospora*.

Regarding the broad spectrum of potential virulence effectors, certain merit further discussion. The *D*. *coniospora* genome is predicted to encode multiple heat-labile enterotoxins. The presence of bacterially-derived enterotoxins in entomopathogenic fungi such as *B*. *bassiana* already presented a conundrum as these species are assumed to lack *per os* infectivity [53]; the same is true for *D*. *coniospora*. Among them, at least 10 are predicted to be secreted (Tables 4 and S6 and S9), so may conceivably be delivered into the host cytoplasm where they would be expected to perturb cellular homeostasis. An alternative explanation is that they may play a role in *D*. *coniospora*’s interactions with other microbes. Competition has already been reported between *D*. *coniospora* and *A*. *oligospora* during nematode infection [110]; antagonistic effects are likely to exist with other species and could rely on enterotoxin production.

Subtilisin-like serine proteases were mentioned several times above. This family has been linked to fungal virulence in nematophagous fungi in some studies (e.g. [8, 73, 111]). Consistent with such a link, subtilisin genes are highly expressed by *A*. *oligospora* and *M*. *haptotylum* during infection of the nematode *C*. *briggsae* [5]. There are 29 genes predicted to encode subtilisins in *D*. *coniospora*. This family is, however, well represented in all the fungal species analysed. For the 11 other species, the number ranges from 12 to 56, with a median value of 31. Since related pathogenic and non-pathogenic species can show the same type of gene expansion, and thus gene number is not correlated with pathogenicity, it has been suggested that the number of serine proteases in a species is related primarily to their role in digestion, whether or not the food source is dead or alive [112].

The genome also contains multiple cysteine-rich secretory protein family genes. These are frequently associated with fungal host adaptation or specialization [67]. As mentioned above, one was found to be preferentially expressed during infection of *C*. *elegans*, despite the poor coverage of the fungal transcriptome. This coverage was in fact remarkably low, despite deep sequencing, with only 0.0011% and 0.0012% of 77 million and 123 million RNAseq reads corresponding to *D*. *coniospora* transcripts from samples of worms infected for 5 and 12 hours, respectively. Clearly, reliable profiling of the *D*. *coniospora* transcriptome at early time points will require the development of methods to enrich fungal mRNA from samples of infected worms. Fortunately, several methods already exist (e.g. [113]); adapting them to this model will require further work.

Such an analysis is likely to be necessary to prioritize the overwhelming number of candidate virulence factors for in-depth functional study. This is particularly true in cases where gene families have expanded and where there is therefore the possibility of functional redundancy. Characterizing expression profiles can allow genes with non-overlapping patterns to be identified; they are less likely to be redundant. While we have shown that *D*. *coniospora* can be readily genetically transformed, this remains a relatively time-consuming process that cannot be implemented on a large scale. Indeed, further work is also needed to develop functional assays to assess the role of the many genes potentially involved in pathogenesis or currently lacking any predictable role. The COPAS Biosort allows the analysis of hundreds of worms each minute. Having the possibility to transform *D*. *coniospora* with a fluorescence protein encoding reporter gene thus provides a non-invasive, non-destructive means to following infection at the level of individual worms at a large scale. It represents a first method of the type that will need to be applied to probe *D*. *coniospora* gene function during its infection of *C*. *elegans*.

We have also given one example of how tagging a protein fluorescently can constitute a first step in its functional characterization. We were able to determine the *in vivo* expression pattern for SapA::dsRed and demonstrate an *in vitro* interaction between it and the host antimicrobial effector SPP-5. We do not yet know whether this interaction mirrors an *in vivo* interaction. We currently favour the idea that SapA::dsRed will be capable of interacting with the host SPP caenopores/saposins that are up-regulated upon infection and thereby inhibit their antimicrobial activity. SPP-5 is very similar in sequence to SPP-2, SPP-6 and SPP-14 that are differentially regulated in *D*. *coniospora* infected worms. Clearly much work remains to be done to clarify the significance of these observations, including establishing which if any of the host SPP caenopores/saposins bind SapA *in vivo*, and determining whether this has any consequence for the progression of the fungal infection. Given the multiplicity of SPP proteins in *C*. *elegans*, this remains a substantial challenge for the future. Clearly, our analysis of the *D*. *coniospora* genome, and the tools we have developed, have opened many avenues for future investigation of this fungus’s antagonistic interaction with *C*. *elegans*.

## Materials and Methods

### *D*. *coniospora* culture and nucleic acid purification

The *D*. *coniospora* strain ATCC 96282 (the kind gift of Hans-Börge Jansson; S1 Text) was either cultured at 25°C on solid Nematode Growth Medium (NGM) in the presence of *C*. *elegans* as previously described [114], or in liquid NGM with Yeast extract (NGMY see S1 Methods) in the absence of nematodes. For the extraction of fungal genomic DNA, roughly 10^9^ fresh spores were inoculated in 100 mL NGMY medium. After 7 days culture, mycelia were harvested by vacuum filtering through a sterile 10 μm nylon membrane. The filter was placed in a 30°C incubator for around 3 h, until the mycelia were dry. The mycelia were then manually ground in a liquid nitrogen-cooled mortar. A 20 mg aliquot was transferred to a 1.5 mL tube and the DNA extracted using the DNeasy Plant Mini Kit (Qiagen), following the manufacturer’s instructions.

To prepare samples for RNA extraction, fresh spores were first collected from 10 cm NGM plates of infected worms as previously described [114] and inoculated in 100 mL of NGMY liquid medium, and either cultured for 4 days before harvesting, or serially cultured (5 x 8 d cultures). For this, a 1 mL aliquot from an 8 d culture was strongly agitated for 5 min to disrupt the tight balls of mycelia and inoculated into fresh 100 mL NGMY liquid medium then cultured for a further 8 days. In both cases, samples were collected by filtering cultures through 0.22 μm Steritop units (Milipore), flash frozen in liquid nitrogen and stored at -80°C. Aliquots of 200 mg of spores or mycelia were dissolved in 1 mL Trizol (Invitrogen) in lysing matrix tubes (MP Biomedicals), homogenized for 20 s at 6 m/s in a FastPrep-24 (MP Biomedicals), incubated on ice for 2 min and homogenized a second time. RNA was purified using a standard protocol [115] and cleaned with an RNeasy mini Kit (Qiagen).

### Library construction and sequencing

MIseq paired-end libraries were prepared from genomic DNA and sequenced on an Illumina MIseq sequencer as 2 x 150 bp paired-end reads following the manufacturer’s standard procedures. For SOLiD sequencing, two mate-paired libraries were prepared from *D*. *conisopora* genomic DNA following the manufacturer’s instructions (Mate-Paired Library Preparation user guide 4460958 Rev.B; Life Technologies, Carlsbad, USA). DNA was sheared to 1.5 kb or 3 kb fragments with Covaris System and Covaris Blue miniTUBES. Following nick-translation for 9.5 min, to generate fragments of < 400 bp, the library was amplified through 14 rounds of PCR. Fragments between 250 and 300 bp were size-selected using 4% acrylamide gel and purified with SOLiD Library Micro Column Purification Kit prior to conversion for analysis with the 5500 WildFire system (Life Technologies, Carlsbad, USA).

cDNA library construction and Illumina sequencing of mRNA from spores and mycelia using an Illumina HiSeq 2000 platform was performed at the Beijing Genomics Institute (Shenzhen, China; http://www.genomics.cn/index.php) using their standard pipeline. More than 48 million 90 bp paired-end reads were obtained from each 200 bp insert library.

### *De novo* genome and transcriptome assembly

MIseq reads were processed with BBDuk software, part of BBMap suite (http://sourceforge.net/projects/bbmap/) to filter out contaminants and low quality reads. The remaining reads were used for *de novo* genome assembly with Velvet [34], SPAdes [35], SOAPdenovo2 [36] and ABySS [37] using standard input parameters except for ABySS for which k values of 64 and 96 were used. The resulting assemblies were then scaffolded using SOLiD mate-paired reads. Only very high quality reads were used for this scaffolding step using SSPACE v2.0 [38] with the k parameter set to 5 (default value).

For use subsequent in gene prediction (see below) a *de novo* transcriptome assembly was performed on the combined sets of reads from the two sequenced libraries (from spores and mycelia) with Trinity [116] using default parameters.

### Optical mapping

A whole genome map of *D*. *coniospora* was generated using the Argus Whole-Genome Mapping System (www.opgen.com). To obtain size-optimized restriction fragments (6–12 kb on average and no fragment larger than 80 kb across the genome) we used Enzyme Chooser (OpGen Inc., Gaithersburg, MD) that led to the selection of *Xba* I. We sorted out 42,622 molecules longer than 200 kb (average 310 kb) used for the assembly, performed with MapSolver software (www.opgen.com). The resulting map contigs were manually validated leading to 9 maps (ranging from 0.58 to 11.3 Mb) with a cumulative size of 31.8 Mb. These were used for comparisons with the *in silico Xba* I digestion profile of the scaffolds obtained after sequencing.

### Functional annotation

Genome annotation was performed using standard open source software. Repetitive elements were mined using RepeatScout version 1.0.5 [42]. A specific repeat library was generated in order to mask the genome with RepeatMasker version 4-0-5 (Smit, Hubley, & Green, RepeatMasker Open-4.0; www.repeatmasker.org). TransposonPSI v08222010 (transposonpsi.sourceforge.net) was used to characterize different types of transposable elements. Non-coding RNA were identified using Rfam scan perl script v1.0 [117] and tRNAscan-SE v1.3.1 [43] for transfer RNAs.

### Gene prediction and annotation

We performed a first round of gene prediction using Augustus [45], trained using the Trinity-derived *D***.** *coniospora* transcripts to predict 8111 protein-coding genes. In a second round, we used Augustus trained using *Fusarium graminearum* (available from http://bioinf.uni-greifswald.de/augustus/) and retained 631 additional predicted proteins that were either (i) conserved, (ii) supported by RNAseq data or (iii) contained a PFAM domain. Inspection of the combined set led to the removal of 9 aberrant proteins (S1 Text), giving a final set of 8733 predicted proteins. Subsequent inspection of the alignment of the unassembled RNAseq reads and the Trinity-derived transcripts to the predicted gene models revealed occasional inconsistencies. Thus as with any first round genome-wide gene prediction and sequence annotation, some errors will need to be resolved in future versions of the genome (see S1 Text).

The set of Augustus-predicted protein-coding genes were annotated using Interproscan [118], release 5.16–55 (data package 55), with Hamap (201511.02), ProDom (2006.1), PIRSF (3.01), PANTHER (10.0), Pfam (28.0), SMART (6.2), Gene3D (3.5.0), Coils (2.2.1), ProSiteProfiles (20.113), TIGRFAM (15.0), PRINTS (42.0), SUPERFAMILY (1.75), and ProSitePatterns (20.113).

The same protocol of InterProScan annotation was performed to annotate the predicted set of proteins from eleven additional fungi. The names indicated are from the NCBI Taxonomy Database; commonly used synonyms and/or NCBI genome assembly accession numbers are in brackets: *Arthrobotrys oligospora* (ADOT00000000.1) [8], *Fusarium graminearum* (*Gibberella zeae*; AACM00000000.2) [119], *Fusarium oxysporum* (AAXH00000000.1) [120], *Hirsutella minnesotensis* (JPUM00000000.1) [9], *Metarhizium acridum* (ADNI00000000.1) [40], *Metarhizium anisopliae* (AZNF00000000.1) [40], *Monacrosporium haptotylum* (*Dactylellina haptotyla*; AQGS00000000.1) [5], *Ophiocordyceps sinensis* (ANOV00000000.1) [41], *Pochonia chlamydosporia* (*Metacordyceps chlamydospora*; AOSW00000000.1) [98], *Tolypocladium ophioglossoides* (*Elaphocordyceps ophioglossoides*; LFRF00000000.1) [64] and *Trichoderma reesei* (*Hypocrea jecorina*; AAIL00000000.2) [121].

SignalP v4.1 [122], TargetP v1.1 [123] and Tmhmm v2.0 [124] were used to predict respectively signal peptide, target peptide and transmembrane domains. A Blast analysis (BLASTP with e value < 10^−5^) versus PHI-base proteins [65] was performed to associate *D*. *coniospora* genes to experimentally verified pathogenicity, virulence and effector genes from fungal, oomycete and bacterial pathogens.

### Phylogenetic analysis

We refined the set of BUSCO-defined orthologues by restricting it to proteins that did not differ by more than 10% in total length across all 12 fungal species. This left us with a set of 97 high-confidence orthologous proteins present in all species. The respective sequences were concatenated were aligned using MAFFT [47] and phylogenetic distances calculated using the maximum likelihood-based method implemented within PhyML [48]. Altering the order of concatenation had no influence on the calculated phylogenetic distances. These analyses were performed within the Mobyle Web environment [125] at http://mobyle.pasteur.fr and the output plotted using the tree drawing engine implemented in the ETE toolkit [126].

### CAZy analysis

Each *D***.** *coniospora* protein model was compared using BLASTP [127] to proteins listed in the CAZy database (www.cazy.org; [128]). Because the e-value depends on the length of the aligned segment (for instance a 30% sequence identity results in widely different e-values, from non-significant to highly significant, if the two aligned proteins are 40, 100, 250 or 500 residues in length), CAZy family assignments rather included examination of sequence conservation (percentage identity over CAZy domain length). Proteins that gave more than 50% identity over the entire domain length of an entry in CAZy were directly assigned to the same family. Proteins with less than 50% identify to a protein in CAZy were all manually inspected and conserved features such as catalytic residues were searched. The variable modular structure of CAZymes was integrated by performing alignments with isolated functional domains [129]. The same methods were used for all fungi that were compared to *D***.** *coniospora*. For clustering of protein families and domains, we used “One minus Pearson correlation” distance matrices within GENE-E (www.broadinstitute.org/cancer/software/GENE-E/).

### Secretome and comparative PFAM analysis

Secretome analysis was carried out as previously described [130] by combining predictions from SignalP v4.1 [122], WolfPSORT [131,132] and NucPred [133]. A WolfPSORT search using mature secreted proteins and model ‘ANIMAL’ was used to determine probable target protein localization in host. For sequence-based clustering, a database consisting of predicted proteomes of *D*. *coniospora*, *H*. *minnesotensis*, *P*. *chlamidosporia*, *M*. *haptotylum*, *A*. *oligospora* and PHIbase v3.6 [65] entries was built. The result of a BLASTP search of this database against itself with an e-value cutoff of 10^−30^ was used as input for clustering with the MCL program in Biolayout Express 3D [134]. Some of these clusters (S11 Table) were projected onto the Circos plot (Fig 1, in red, purple and green, respectively): OG5_126718 as putative nonribosomal peptide synthetases; OG5_127207 (serine carboxypeptidase S28), OG5_138644 (deuterolysin metalloprotease (M35) family), OG5_137388 (PA domain; subtilase family), OG5_128249 (subtilase family), OG5_149879 (serine carboxypeptidase), together with orthoMCL-defined paralogGroup 11 (subtilisin-like serine protease; S6 Table) as diverse proteases; paralogGroups 1, 4, 9 and 17 as enterotoxin-like proteins. Clusters that did not contain predicted secreted proteins from any of the 5 fungal species were discarded. Correspondence analysis was performed using the FactomineR package in R.

TargetP v1.1 [123] was used to determine probable protein localization, using mature proteins for secreted proteins or full-length sequences otherwise. A consensus predicted localization was derived using the following rules: TargetP predictions with reliability < = 3 only were considered; NLS were considered if predicted by NLStradamus and with a NucPred score <0.6; PredGPI predictions were considered if probability > = 90. PFAM domains were identified through a search against PFAM 28.0 database using gathering thresholds.

### Gene expression analysis

For samples from mycelia and spores, Illumina paired-end (2 x 90 bp) RNAseq reads were aligned to the genome assembly using STAR [135]. Reads were assigned to the 8733 gene models using the htseq-count script within HTSeq [136]. To establish lists of differentially regulated genes, we used previous described methods [74] and retained the genes that were commonly defined by both. For samples from *C*. *elegans* infected with *D*. *coniospora*, the unaligned reads (640069 and 237794 reads from 5 and 12 h samples, respectively; kindly provided by LaDeana Hillier) from a previous RNAseq analysis [74] were aligned and assigned to gene models as above.

### Protoplast preparation and fungal transformatn

N2 worms at the L4 stage were infected with fungal spores as described [114] on NGMY plates spread with the *E*. *coli* strain OP50 and incubated for 24 h, then transferred into NGMY liquid medium and cultured for another 30 h. Mycelia were collected by filtration as above and protoplasts prepared and transformed using polyethylene glycol (PEG)/CaCl_2_-mediated DNA uptake as described [137], using expression vectors (see S1 Methods) containing a hygromycin selection marker, derived from pPK2*hphgfp* [78], a kind gift from Martijn Rep (Swammerdam Institute for Life Sciences, Amsterdam). Transformants were selected for antibiotic resistance on medium containing 15 μg/ml hygromycin and screened for fluorescence.

### Spore protein lysates and pull-down assays

Worms were infected on NGM plates with spores from SapA::DsRed expressing fungus. After 15 to 30 day culture at 25°C, spores were harvested in 50 mM NaCl as previously described [114]. They were extensively washed in cold 50 mM NaCl, pelleted and resuspended in an equal volume of lysis buffer (50mM Tris-Cl, pH 7.5, 100mM NaCl, 3 mM MgCl2, 0.5% Triton X-100, protease inhibitors (Complete, Roche), 5% glycerol) and flash-frozen. They were then sonicated on a high setting for 7 minutes (30 sec on and 30 sec off; Bioruptor, Diagenode,) and vortexed for 5 min with acid-washed glass beads (Sigma). The supernatant from a high-speed centrifugation was used as a whole protein extract for pull-down assays. For this, 800 μg of protein extracts were incubated with 20 μg of purified His-tagged SPP-1, SPP-5, or SPP-12 (the generous gift of M. Leippe, Kiel university), for 2 h. Preformed complexes were then immunoprecipitated with anti-dsRed/RFP agarose beads (Chromotek, RFP-Trap), at 4°C, overnight. As a binding control, preformed complexes were incubated under identical conditions with blocked matrix beads. After incubation, beads were washed three times in lysis buffer and three times in wash buffer (25 mM Tris-HCl, pH 7.5, 150 mM NaCl, 0.1% NP-40, 1 mM MgCl_2_). After a final wash (1 mM Tris-Cl, 150 mM NaCl and 1 mM MgCl_2_) beads were eluted in SDS-PAGE loading buffer. Samples were then resolved on a 4–12% gradient denaturating PAGE gel and transferred onto a membrane. The blot was probed with anti-His antibody (Upstate, H8 clone) or anti-dsRed/RFP antibody (Rockland, Inc). Blots were visualized using enhanced chemiluminescence (SuperSignal West Pico, Pierce).

### Nucleotide sequence accession numbers

MIseq reads used for *de novo* genome assembly and Hiseq reads used for RNAseq *de novo* have been deposited at SRA under accession numbers SRX883538 and SRX969055, respectively. The Whole Genome Shotgun project has been deposited at DDBJ/ENA/GenBank under the accession JYHR00000000. The version described in this paper is version JYHR01000000.

## Supporting Information

S1 Table Descriptive statistics of the different assemblies before and after SSPACE scaffolding. Correspondence of the 9 optical maps (named as chromosomes) to the scaffolds from Velvet (kmer = 63), SOAPdenovo (kmer = 63), Spades (kmer = 127), and ABySS (kmer = 64 and 96), before and after scaffolding with SSPACE. Two values are provided, which correspond to the number of scaffolds localized on each optical map and the total coverage in Mb. The total number of mis-assemblies observed within each analysis is also indicated. ND: not determined.
(XLSX)

S2 Table Comparative analysis of transposable elements in the genome of *D*. *coniospora* and of 11 other fungal species. The sources for the genome sequences used in this analysis are given in the Materials and Methods.
(XLSX)

S3 Table Comparative analysis of tRNAs in the genome of *D*. *coniospora* and of 11 other fungal species. (XLSX)

S4 Table Species and sequences used for phylogenetic analysis. (XLSX)

S5 Table InterproScan analysis of the 8733 proteins predicted from the *D*. *coniospora* genome. The first sheet presents the results of an InterproScan analysis of the predicted proteome, complemented with dedicated BLASTP searches against UniprotKB and PHI-base databases. The second gives the sequences of 31 predicted proteins longer than 1000 amino acids, but without any InterproScan annotation. The third sheet gives a summary of the BLASTP analysis against the NCBI non-redundant set of proteins for the 31 sequences on the previous sheet. The results for the predicted proteins are split into 3 groups: with a homologue (only top 10 hits shown), with only remote similarity to other proteins (all hits shown), and with no similarity to any protein in the current NCBI non-redundant protein sequence database.
(XLSX)

S6 Table OrthoMCL analysis of the 8733 proteins predicted from the *D*. *coniospora* genome. The first sheet lists the species present in OrthoMCL, with the frequency of top hits per species. The second sheet indicates the top OrthoMCL hit for the 7092 *D*. *coniospora* proteins assigned to an OrthoMCL cluster. The third sheet gives the number of predicted proteins in each OrthoMCL cluster, with corresponding PFAM domain annotation (from S5 Table) for those with more than 3 members. The fourth sheet lists the members of the clusters determined by OrthoMCL to be specific to *D*. *coniospora* (i.e. not represented in the 150 species in the first sheet). The fifth sheet gives the CLUSTAL Omega alignments for the 15 predicted proteins in the OrthoMCL paralogous group 1 (from fourth sheet).
(XLSX)

S7 Table Comparative analysis of the occurrence of PFAM domains in the 8733 proteins predicted from the *D*. *coniospora* genome and in the predicted proteins from 11 other fungal species. Only PFAM domains present in at least one species are shown. The first sheet shows all the data. The second and third, extracted from the first, shows PFAM domains absent and present in *D*. *coniospora*, respectively.
(XLSX)

S8 Table Detailed analysis of *D*. *coniospora* CAZy proteins and comparative analysis with the predicted CAZy proteins from 10 other fungal species. The first sheet indicates the classification of the predicted 240 CAZy proteins in *D*. *coniospora*, with notes from manual annotation. The following sheets show the occurrence of different CAZy family proteins in *D*. *coniospora* and 10 other fungi. Only CAZy families present in at least one species are shown.
(XLSX)

S9 Table Predicted *D*. *coniospora* proteins involved in the production of secondary metabolites. The first sheet lists the proteins identified by SMURF analysis as ‘backbone’; the second lists the proteins that putatively form part of a functional cluster.
(XLSX)

S10 Table Analysis of hits in the PHI-base database for the 8,733 proteins predicted from the *D*. *coniospora* genome. The first sheet gives the number of times a given PHI-base entry was returned as the top hit for a *D*. *coniospora* protein (indicated in the column ‘Occurrence’; data derived from the column ‘PHIBASE’ in S5 Table). The second sheet lists the PHI-base entries for those hit by 5 or more predicted *D*. *coniospora* proteins. Certain indicated functional categories are highlighted in colour. The third sheet is an extract of the InterproScan data in S5 Table for all the *D*. *coniospora* proteins listed in the second sheet, and the following 7 sheets are extracts for the proteins corresponding to the PHI-base entries hit at least 10 times.
(XLSX)

S11 Table Details of the secretome analysis for the 670 *D*. *coniospora* predicted with high confidence to be secreted. The first sheet lists the *D*. *coniospora* proteins predicted to be secreted, together with the scores from the analysis programs, and annotations taken from S5 Table. The second sheet gives the data sources for the comparative analysis. The last sheet shows the results of sequence based clustering of proteins from five nematophagous fungal species. Only clusters including at least one predicted secreted protein are considered.
(XLSX)

S12 Table RNAseq analysis of transcripts from spore and mycelial samples. The first sheet gives the read counts for each gene from the 2 different samples. The 50 most highly expressed genes for each sample are highlighted. In the next 2 sheets they are listed together with annotations taken from S5 Table. The fourth and the fifth sheets list the genes assigned to the over-represented category in spores and in mycelia, respectively, together with annotations taken from S5 Table. On the fourth sheet, neighbouring genes are highlighted in yellow. The sixth and seventh list their respective constituent PFAM domains, with a score that reflects the confidence of the assignment. Seven domains are found in both lists (PF00005, PF00083, PF00172, PF00501, PF05730, PF07690, PF13193).
(XLSX)

S13 Table RNAseq analysis of *D*. *coniospora* transcripts from samples extracted from infected *C*. *elegans*. The first sheet gives the raw read counts for genes covered by at least one RNAseq read from the 2 samples, listed by name and total read counts. The second sheet lists the genes covered by at least 3 reads, gives the read counts for the samples from spores and mycelia (data from S12 Table; genes in the top 15^th^ percentile highlighted) and includes different functional annotations (from S5 Table).
(XLSX)

S1 Fig RADAR analysis [54] reveals the repeated structure in the sequence of g8068.t1, a 1045 a.a. protein from OrthoMCL-defined paralogous group 2 (see S6 Table). (PDF)

S2 Fig Photomicrographs of mycelial (A, B, C) or spore (D) preparations of *D*. *coniospora*, taken shortly before processing for RNA extraction. (A) Under the conditions of liquid culture used, *D*. *coniospora* forms compact balls of up to several mm in diameter. (B, C) At a higher magnification, it can be seen that the mycelia are devoid of spores and the fusion of hyphae can be clearly observed (white arrows). (D) While the majority of spores have started to germinate (red arrows), some have not (white arrows). A smaller proportion is not mature, lacking the adhesive bud (yellow arrows). Scale bars (white) in C and D, 10 μm.
(PDF)

S3 Fig PCR-based verification of the insertion of a hygromycin-resistance expression cassette into the *Dso* locus. The top part of the figure shows the position of PCR primers relative to the genomic and recombinant DNA sequences. The 2 tables indicate the expected sizes and occurrences of PCR amplicons. The bottom part of the figure shows that the expected bands are obtained from the wild-type (WT) and knocked-in strain (Dso).
(PDF)

S4 Fig (A) PCR products from reverse-transcribed mRNA corresponding to the *D*. *coniospora* saposin A-domain protein-encoding gene g3895.t1 (SapA) and the actin gene g2551.t1 (Actin) from mycelia and at the indicated times post-infection (p.i.). The size markers in the outside lanes are, from top to bottom, 300, 200 and 100 bp. (B) Clustal multiple alignment of infection-induced saposin proteins (in bold) and those used for to assay for a possible interaction between a host saposin and the fungal SapA protein.
(PDF)

S1 Text Contains comments on the quality of the genome sequence and gene annotation, as well as a description of the isolation history of ATCC 96282 and its derivatives. (PDF)

S1 Methods Contains details of media, plasmid constructions and primer sequences. (DOCX)
