# Supplementary material for: Comparative Genomic Analysis of Drechmeria coniospora Reveals Core and Specific Genetic Requirements for Fungal Endoparasitism of Nematodes
Source: PLoS Genet. 2016 May 6;12(5):e1006017. doi: 10.1371/journal.pgen.1006017 (PMC4859500; doi:10.1371/journal.pgen.1006017)
Supplement: S1 Fig — (PDF) [file pgen.1006017.s014.pdf]

```
1-36         .MHIQLRND..R..Y.RNGY.grhcnPDN..YGTGSGYDDDSAAA
37-74        WLHIQLRNEHRR..Y.RSCW..whhdPDD..GRTGTGHDDDPAAA
75-111       RMQFRLRNECYR....NGYG..whhdPDD..HGTGSRHDDDSAAA
112-149      WLHIQLRDEHRR..Y.RSCW..whhdPDN..GRPGPRYKYDSAAA
150-186      WMYFRLRNE..R..Y.RNCY.eryrdPDD..YGTGSGYEYDGAAA
187-223      WMHIQLRDE..R..Y.RDCY.rrlydPDD..YGTGSGYEHDGTAA
224-261      WMHIQLRNKHYR..Y.RSCW..whydPDD..GRPGPGYKYDSAAA
262-298      RVYFRLCNECYR....NGYG..rhrnPDD..YGSGSRHDDDSAAT
299-335      WLYIQLRDE..R..Y.RDCY.rrlydPDD..YGTGSGHEHDGTAA
336-373      WMHIQLRNKHYR..Y.RSCW..whydPDD..GRPGPRYKYDSAAA
374-410      RVYFRLCNE..R..Y.RNCY.grlndPDD..YGTGSGYEHDSTAA
411-448      WMHIQLRNEYYR..Y.RSCW..whydPDD..GRPSPRYKYDSAAA
449-485      WVYFRLCNE..R..Y.RNCY.rrlydPDD..YGTGSGITTQTTTG
505-541      RVHCRLRDE..R..Y.RDGY.gwlydPDD..YGTGSGYEYDRTTA
542-579      WMHIQLHNEHYR..Y.RSCW..whydPDD..YGTGSGHEHDGTAA
580-619      WMHIQLRNEHYR..Y.RSCW..whydPDDddYGTGSGYEHNGTAA
620-656      WMYIQLRNEHYRngYgRHC......nPDD..YGTGSRHDDDCAAA
657-693      WMHIQLRDE..R..Y.RDCY.rrlydPDD..YGTGSGYKYNSATA
694-730      RVYIQLRNEHYRngYgRHC......nPDD..YGTGSRHDDDCAAA
731-767      WMHIQLRDE..R..Y.RDCY.rrlydPDD..YGTGSGYEHDGTAA
768-805      WMHIQLRNEHYR..Y.RSCW..whydPDD..YGTGSGHEYDGTAA
806-845      WMYIQLRNEHYR..Y.RSCWwhydsdDDD..YGTGSGYEHDGTAA
846-882      WMHIQLRNEHYRngYgRHC......nPDD..YGTGSRHDDDCAAA
883-914      WMHIQLRDE..R..Y.CDCY.rrlydPDD..YGTGSGYEH.....
916-931      ..........................DD..YGTGSGHEHDGTAA
932-969      WMHIQLPNEHYR..Y.RSCW..whydPDD..YGTGSGYEHDGTAA
970-1007     WMHIQLRNEHYR..Y.RSCW..whydPDD..YGTGSGYEHDGTAA
1008-1045    WMHIQLRNEHYH..N.RSCW..whydPDD..YGTGSGYKHDSTAA
```
