# Supplementary figures and images for: Comparative Genomic Analysis of Drechmeria coniospora Reveals Core and Specific Genetic Requirements for Fungal Endoparasitism of Nematodes
Source: PLoS Genet. 2016 May 6;12(5):e1006017. doi: 10.1371/journal.pgen.1006017 (PMC4859500; doi:10.1371/journal.pgen.1006017)

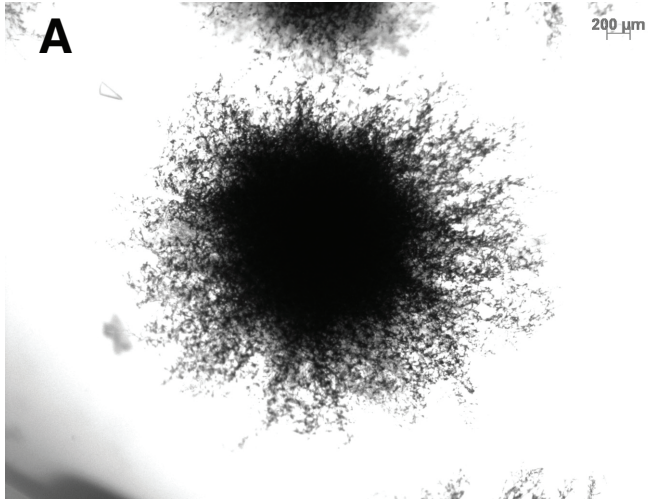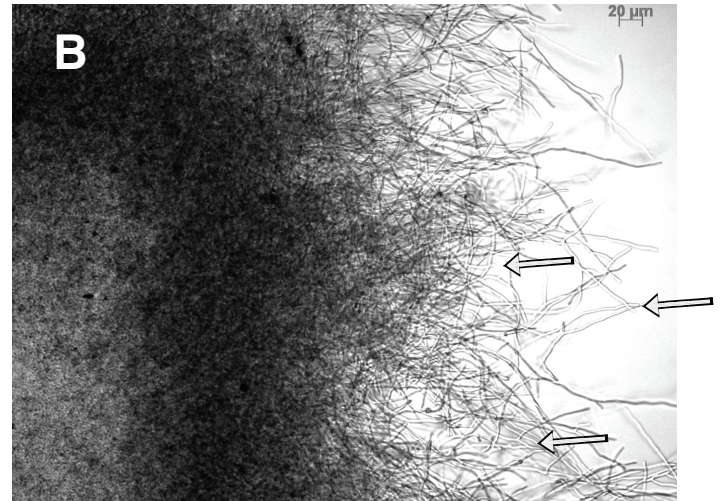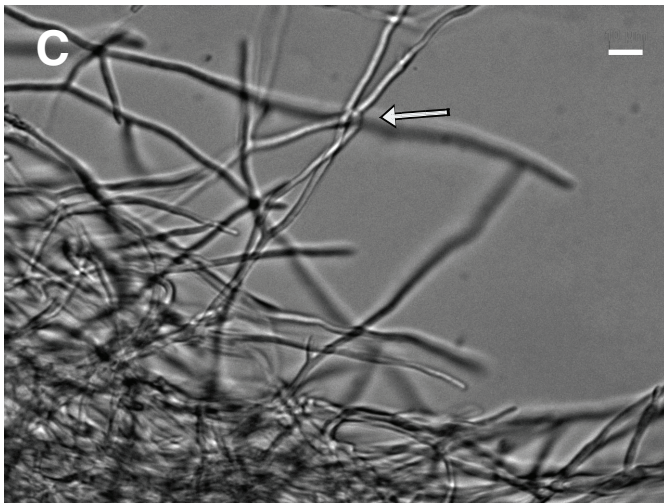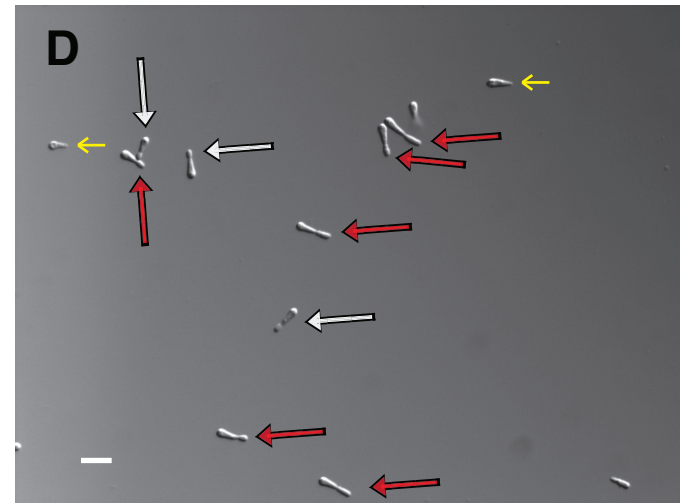

Supplement: S2 Fig — (A) Under the conditions of liquid culture used, D. coniospora forms compact balls of up to several mm in diameter. (B, C) At a higher magnification, it can be seen that the mycelia are devoid of spores and the fusion of hyphae can be clearly observed (white arrows). (D) While the majority of spores have started to germinate (red arrows), some have not (white arrows). A smaller proportion is not mature, lacking the adhesive bud (yellow arrows). Scale bars (white) in C and D, 10 μm. (PDF) [file pgen.1006017.s015.pdf]
