# Supplementary material for: Comparative Genomic Analysis of Drechmeria coniospora Reveals Core and Specific Genetic Requirements for Fungal Endoparasitism of Nematodes
Source: PLoS Genet. 2016 May 6;12(5):e1006017. doi: 10.1371/journal.pgen.1006017 (PMC4859500; doi:10.1371/journal.pgen.1006017)

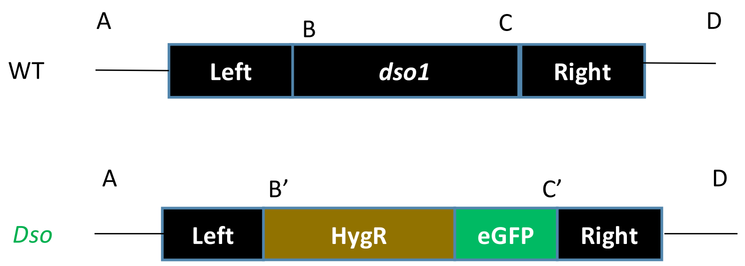

	Primer	Primer sequence
A	JEP2267	CGAGTCCAGCGACTTGCGAC
B	JEP2674	CGCACGCCGCGGCTGGCTTC
B’	JEP2266	CTTGTTGATGTACGGAGCACCC
C	JEP2675	CACATCTGGAGTACGGAAGG
C’	JEP2269	GCATGGACGAGCTGTACAAG
D	JEP2268	GTACCAGGTACCTACCCCTCC
HygR F	JEP2162	ATGCCTGAACTCACCGCGAC
HygR R	JEP2216	TTCCTTTGCCCTCGGACGAG

Primer pair	Expected size	Amplifies from WT	Amplifies from *Dso* knock-in
AB	1.6 kb	Yes	No
AB’	1.6 kb	No	Yes
CD	1.6 kb	Yes	No
CD’	1.6 kb	No	Yes
HygR F/R	1 kb	No	Yes

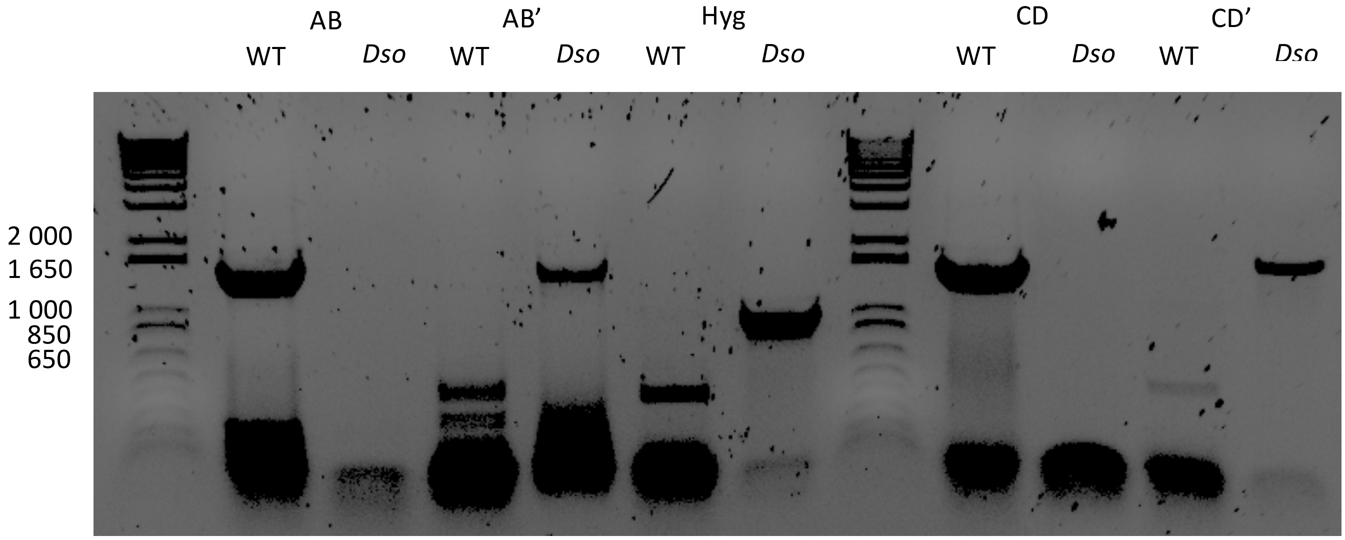

Supplement: S3 Fig — The top part of the figure shows the position of PCR primers relative to the genomic and recombinant DNA sequences. The 2 tables indicate the expected sizes and occurrences of PCR amplicons. The bottom part of the figure shows that the expected bands are obtained from the wild-type (WT) and knocked-in strain (Dso). (PDF) [file pgen.1006017.s016.pdf]
