# Supplementary material for: Comparative Genomic Analysis of Drechmeria coniospora Reveals Core and Specific Genetic Requirements for Fungal Endoparasitism of Nematodes
Source: PLoS Genet. 2016 May 6;12(5):e1006017. doi: 10.1371/journal.pgen.1006017 (PMC4859500; doi:10.1371/journal.pgen.1006017)
Supplement: S4 Fig — (A) PCR products from reverse-transcribed mRNA corresponding to the D. coniospora saposin A-domain protein-encoding gene g3895.t1 (SapA) and the actin gene g2551.t1 (Actin) from mycelia and at the indicated times post-infection (p.i.). The size markers in the outside lanes are, from top to bottom, 300, 200 and 100 bp. (B) Clustal multiple alignment of infection-induced saposin proteins (in bold) and those used for to assay for a possible interaction between a host saposin and the fungal SapA protein. (PDF) [file pgen.1006017.s017.pdf]

A

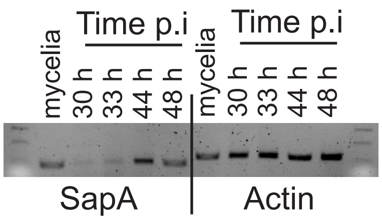

B

```
WBGene00004998|F08F1.6|SPP-13    MQSLQLLTFVLIALMVSVTFAEIIQKGKLAKHGMLKENKPNCLLTRSRLGCTCTTCKEIV
WBGene00004999|K09F5.3|SPP-14    MKT----TFCLLA-LVAVATAL-------------------ALPSQQDNAMSCLMCEVGV
WBGene00004991|T08A9.10|SPP-6    ------------------MTCM-------------------TGLR-QRNALDCEMCKLAV
WBGene00004987|T08A9.12|SPP-2    MKT----VLVLAA-VAAVALCV-------------------VLPK-ERSSLGCQMCELAV
WBGene00004990|T08A9.9|SPP-5     MKT----LLVLAV-LVAVASGL-------------------AIPE-NRSALSCQMCELVV
WBGene00005000|C48E7.10|SPP-15   -MNYLIL---LA--FLAITLTVSNV-------------EGAKKMHSDTSKPLCGLCVNVV
WBGene00004986|T07C4.4|SPP-1     -MTRILPCLFLV--LLAAAPLLANP-------------AN-PLNLKKHHGVFCDVCKALV
WBGene00004997|T22G5.7|SPP-12    MFSK--TVVVLL--MVVPAISLAQP-------------AS-PLVLKKSHGAFCHLCEDLI
                                                                              .     *  *   :

WBGene00004998|F08F1.6|SPP-13    NFTRMLILNHVPE-EQEVMEKVCYRIFGDDKKKESFCEELIKEELPDIIKYVRNHLEPKQ
WBGene00004999|K09F5.3|SPP-14    RAAENPADREAHT-VEDKFDAECKKELGIIPFAEKECEKYGNSKLDPIINELEGGTAPED
WBGene00004991|T08A9.10|SPP-6    KSADGDADKDTND-IKKDFDAKCKKAFHSIQFAPRECEHYVDKKLDPIIKELESGTSPKD
WBGene00004987|T08A9.12|SPP-2    KTYDGSADKDVTS-IKKDFDTECKKLFHAIPFAPQECEHYVNEKLDPIIKELESGTAPKD
WBGene00004990|T08A9.9|SPP-5     KKYEGSADKDANV-IKKDFDAECKKLFHTIPFGTRECDHYVNSKVDPIIHELEGGTAPKD
WBGene00005000|C48E7.10|SPP-15   KQLDQVLEHGGD--IEAAVDKFCKEDVP--SFMVDMCEKVIEKNLEYIINKLKDHEEADK
WBGene00004986|T07C4.4|SPP-1     EGGEKVGDDDLDAWLDVNIGTLCWTML---LPLHHECEEELKKVKKELKKDIENKDSPDK
WBGene00004997|T22G5.7|SPP-12    KDGKEAGDVALDVWLDEEIGSRCKDFG----VLASECFKELKVAEHDIWEAIDQEIPEDK
                                 .              .  .   *             * .  .     : . :      ..

WBGene00004998|F08F1.6|SPP-13    ACAKFC---------------
WBGene00004999|K09F5.3|SPP-14    VCKKLKEC-------------
WBGene00004991|T08A9.10|SPP-6    VCTKLGECS------------
WBGene00004987|T08A9.12|SPP-2    VCKKLGECPK-----------
WBGene00004990|T08A9.9|SPP-5     VCTKLNECP------------
WBGene00005000|C48E7.10|SPP-15   ICTDILLCRTPKQYYFLETQK
WBGene00004986|T07C4.4|SPP-1     ACKDVDLC-------------
WBGene00004997|T22G5.7|SPP-12    TCKEAKLC-------------
                                  *
```
