# Supplementary material for: Comparative Genomic Analysis of Drechmeria coniospora Reveals Core and Specific Genetic Requirements for Fungal Endoparasitism of Nematodes
Source: PLoS Genet. 2016 May 6;12(5):e1006017. doi: 10.1371/journal.pgen.1006017 (PMC4859500; doi:10.1371/journal.pgen.1006017)
Supplement: S1 Text — (PDF) [file pgen.1006017.s018.pdf]

Genome assembly, gene prediction and annotation.

As is standard practice, as far as possible, we adopted automated pipelines for data analysis. Judged against published fungal genomes, the genome of *D. coniospora* is clearly of a very high quality in terms of coverage and accuracy. We note, however, that these procedures did introduce a number of artefacts that will need correction in future versions of the annotated genome. Among the minor anomalies, we note the following:

As described in the main text, Scaffold43 was identified as being of mitochondrial origin. A more comprehensive investigation of this scaffold with TBLASTX against a database of mitochondrial genes identified three mitochondrial-specific ATP synthases (*atp6*, *atp8*, *atp9*), one cytochrome b (*cob*), two cytochrome oxidases (*cox1* and *cox2*), six subunits of NADH dehydrogenase (*nad1*, *nad2*, *nad3*, *nad4*, *nad4L*, *nad5*), and one *rps5* ribosomal protein. Fungal genomes generally have a conserved structure, with 14 protein-encoding genes arranged in the order *nad2*, *nad3*, *atp9*, *cox2*, *nad4L*, *nad5*, *cob*, *cox1*, *nad1*, *nad4*, *atp8*, *atp6*, *cox3* and *nad6*. The absence of genes for *cox3* and *nad6* from Scaffold43 suggested a truncation of the sequence. Genes for *cox3* and *nad6* were found next to each other (coordinates 2196045-2196848 and 2197076-2197738) within the sequence of omap49267b (a scaffold of 10013798 bp), probably the result of an assembly error.
We also observed that a short DNA sequence was duplicated on 2 separate unmapped scaffolds. Specifically, scaf62 (5655 bp) was found to be 99% identical to scaf63 (5653 bp). Further, the 5' 3334 bp of scaf62 matches perfectly the start of both scaf59 and scaf60, while the 3' 1939 bp matches a sequence within omap3956. The most plausible explanation for all these observations is an error in automated assembly. To avoid biasing slightly our analyses, the 8 supernumerary copies of the proteins predicted for these regions were removed from an original Augustus-derived set of 8742 proteins, together with a single protein that had been predicted despite its lack of a 5' ATG. There may be other isolated examples of misassembly leading to artificial duplication of genes. For example, we note that the 3 "glycine rich protein" (PF12810) domain genes referred to in the main text (fg9098.t1, fg9056.t1, fg8010.t1) on omap6109, scaf41 and scaf68 are 100% identical at the DNA level, and so could be the result of very recent gene duplication events but might be the consequence of imperfect assembly. At the level of individual proteins, a detailed analysis of *D. coniospora* CAZy proteins (Supplementary Table S8) suggested that 16/240 (<7%) of predicted gene models might not be entirely correct. Rectification of these different types of errors will require painstaking re-analysis and expert curation and will be included in a future version of the genome.

With regards 3rd party annotation, among the domains unique to *D. coniospora*, PF02609 (Exonuclease VII small subunit) was present in g4632.t1. This is a clear homolog of the highly conserved protein Rad50 (Supplementary Table S5), a protein devoid of a PF02609 domain. Further inspection showed the domain assignment for g4632.t1 to be very tenuous, and due to the insertion of a hexapeptide sequence "ERYRQD" within the otherwise well-conserved 200 residue-long AAA (PF13476) domain. The PF02609 domain is therefore not discussed further in the main text.

Note added in proof.

While the revised version of this article was in review, a genome sequence for *D. coniospora* strain ARSEF 6962 was reported [1]. As explained below, these strains were derived from a common isolate. Despite global concurrence between the 2 sequences and between the sets of predicted genes, preliminary comparisons indicate an unexpectedly high level of sequence divergence, including an elevated level of single nucleotide polymorphisms, as well as multiple indels, including some of up to several kb in length. Determining whether these reflect errors in sequencing and/or assembly for one or other sequence, or true molecular divergence will require targeted investigation in the future.

One striking difference concerns the number of predicted chromosomes, 9 (this study) versus 3 [1]. As optical mapping was used in both cases, a detailed comparison of the respective data will be required to provide an explanation for this discrepancy.

Another notable difference is the prediction from the ARSEF 6962 sequence of a protein containing a MATα (PF04769) domain. This lead Zhang *et al.* to speculate on the existence of a cryptic sexual cycle for *D. coniospora* and to suggest that it might be a heterothallic species [1]. Again, this important point will require clarification.

The strain used in the current study was originally provided to us in 1998 by Hans-Börje Jansson, then at Lund University, Sweden. At the time, he wrote that it was the strain “called "ny" ATCC# 96282 used in the JON paper [Journal of Nematology (26:430; 1994)]”. In the “JON paper”, one finds mention of “The fungus *Drechmeria coniospora*, designated isolate no. 5 by Jansson (9)”, with reference 9 being:

Jansson HB. Adhesion to Nematodes of Conidia from the Nematophagous Fungus *Drechmeria coniospora*. Journal of General Microbiology. 1993;139:1899-906.

The strain was deposited in the CBS-KNAW culture collection by Jansson and is held under the name CBS 615.82, and is in the ARSEF collection as strain 2468. To summarise, ATCC 96282 is variously called, “ny”, “isolate no. 5”, “CBS 615.82” and “ARSEF 2468”. The strain ARSEF 6962 is described in the ARSEF catalogue as a “reisolation of ARSEF 2468”, and should therefore be a re-isolation of ATCC 96282.

It is important to note that the strain provided to us by Jansson had been in almost continuous culture in our laboratory, passaged through infection of *C. elegans*, for some 15 years before a sample of its DNA was extracted for genome sequencing.

1. Zhang L, Zhou Z, Guo Q, Fokkens L, Miskei M, Pocsi I, et al. Insights into Adaptations to a Near-Obligate Nematode Endoparasitic Lifestyle from the Finished Genome of *Drechmeria coniospora*. Sci Rep. 2016;6:23122. doi: 10.1038/srep23122. PubMed PMID: 26975455; PubMed Central PMCID: PMCPMC4792172.
