# Supplementary material for: Comparative Genomic Analysis of Drechmeria coniospora Reveals Core and Specific Genetic Requirements for Fungal Endoparasitism of Nematodes
Source: PLoS Genet. 2016 May 6;12(5):e1006017. doi: 10.1371/journal.pgen.1006017 (PMC4859500; doi:10.1371/journal.pgen.1006017)
Supplement: S1 Methods — (DOCX) [file pgen.1006017.s019.docx]

**Supplementary Methods**

**Media**

Nematode Growth Medium (NGM) [[1](#_ENREF_1)]:

| NaCl | 3 g |
| --- | --- |
| BactoPeptone | 2.5 g |
| BactoAgar | 20 g |
| 5 mg/mL cholesterol in EtOH | 1 mL |
| 1 M MgSO_4_ | 1 mL |
| 1 M CaCl_2_ | 1 mL |
| 1 M KPO_4_ pH 6.0 | 25 mL |

Nematode Growth Medium with Yeast extract (NGMY). For 1 litre:

| NaCl | 3 g |
| --- | --- |
| BactoPeptone | 2.5 g |
| Yeast Extract | 20 g |
| 5 mg/mL cholesterol in EtOH | 1 mL |
| 1 M MgSO_4_ | 1 mL |
| 1 M CaCl_2_ | 1 mL |
| 1 M KPO_4_ pH 6.0 | 25 mL |
| 100 mg/mL Ampicilin | 1 mL |
| Gentamycin | 1 mL |

Solid NGMY medium was made following the same recipe but with the addition of 20 g/l BactoAgar.

**Plasmid construction**

To construct the pLH4237 plasmid containing *hphgfp* under the control of the *D. coniospora* beta-tubulin promoter (from g807.t1), the backbone, *hphgfp* and *trpC* terminator sequences from pPK2*hphgfp* [[2](#_ENREF_2)] were amplified with primers JEP2162/JEP 2163 and fused with *D. coniospora* beta-tubulin promoter (PCR amplified from genomic DNA with JEP2160/JEP2161) via Gibson assembly [[3](#_ENREF_3)].

To construct the plasmid targeting *Dso* (g1469.t1), the gene together with 1 kb 5’ and 3’ flanking arms were PCR amplified from genomic DNA (using primers JEP2239/JEP2240) and cloned into pGEM-T Easy (Promega). This plasmid was then used as a template to amplify the 5’ and 3’ flanking arms and the pGEM-T backbone sequence (using primers JEP2237/JEP2238) and ligated to the beta-tubulin promoter *hphgfp* sequence from pLH4237 (PCR amplified using primers JEP2242/JEP 2243) by Gibson assembly to give pLH4256.

To make the SapA::dsRed reporter, we PCR amplified 4 separate DNA fragments: (i) the *sapA* coding region without the stop codon and with 1 kb of sequence upstream of the predicted ATG, from genomic DNA with primers JEP2594/JEP2596. (ii) The coding sequence for dsRed from the plasmid pLH4238 with JEP2592/JEP2593. (iii) The beta-tubulin promoter, *hphgfp* and *trpC* terminator from pLH4237, with JEP2242/JEP2591 (iv) The 5’ and 3’ flanking arms of *Dso* and the pGEM-T backbone sequence with JEP2237/JEP2238. The fragments were joined by Gibson assembly to give pLH4244.

**Primer sequences**

JEP2160 GATATCGAGCTCGGTACCCGGCTAGGGTGCTCCGTACATC

JEP2161 GTCGCGGTGAGTTCAGGCATCTATATCGAAGAAGGAAACTCAAG

JEP2162 ATGCCTGAACTCACCGCGAC

JEP2163 CCGGGTACCGAGCTCGATATC

JEP2237 TGAAGGATGAGCGACGGCGC

JEP2238 CATCGTCCCTTGCTCGCACAG

JEP2239 AGCTGTGCTCCGTGTGTTGT

JEP2240 CTCCGCCAGAAATGCACCGT

JEP2242 CTGTGCGAGCAAGGGACGATGTAGGGTGCTCCGTACATC

JEP2243 GCGCCGTCGCTCATCCTTCACTTGTACAGCTCGTCCATGC

JEP2592 GGGCCCATGGCCTCCTCCGA

JEP2593 GCGCCGTCGCTCATCCTTCATCAGTTGGAATTCG

JEP2594 CTCCACTCGACCTGCAGGTCGAATGGCCCTCCAAGTTG

JEP2596 GGAGGAGGCCATGGGCCCTGTCGTGGTAGCAGCCGACG

JEP2242 CTGTGCGAGCAAGGGACGATGTAGGGTGCTCCGTACATC

JEP2591 CTGCAGGTCGAGTGGAGATG

1. Stiernagle T. Maintenance of *C. elegans*. http://www.wormbook.org: The *C. elegans* Research Community ed; 2006. Available from: http://www.wormbook.org.

2. Michielse CB, van Wijk R, Reijnen L, Cornelissen BJ, Rep M. Insight into the molecular requirements for pathogenicity of *Fusarium oxysporum* f. sp. *lycopersici* through large-scale insertional mutagenesis. Genome biology. 2009;10(1):R4. Epub 2009/01/13. doi: 10.1186/gb-2009-10-1-r4. PubMed PMID: 19134172; PubMed Central PMCID: PMC2687792.

3. Gibson DG, Young L, Chuang RY, Venter JC, Hutchison CA, 3rd, Smith HO. Enzymatic assembly of DNA molecules up to several hundred kilobases. Nature methods. 2009;6(5):343-5. Epub 2009/04/14. doi: 10.1038/nmeth.1318. PubMed PMID: 19363495.
